# Radiation Effects on Uranyl Tetrachloro Coordination Compounds: Impact of Lattice Water

**DOI:** 10.1021/acs.inorgchem.5c00693

**Published:** 2025-05-07

**Authors:** Harindu Rajapaksha, Samantha J. Kruse, Jay A. LaVerne, Sara E. Mason, Tori Z. Forbes

**Affiliations:** † Department of Chemistry, University of Iowa, Iowa City, Iowa 52242, United States; ‡ Department of Physics and Astronomy, University of Notre Dame, Notre Dame, Indiana 46556, United States; § Center for Functional Nanomaterials, Brookhaven National Laboratory, Upton, New York 11973, United States

## Abstract

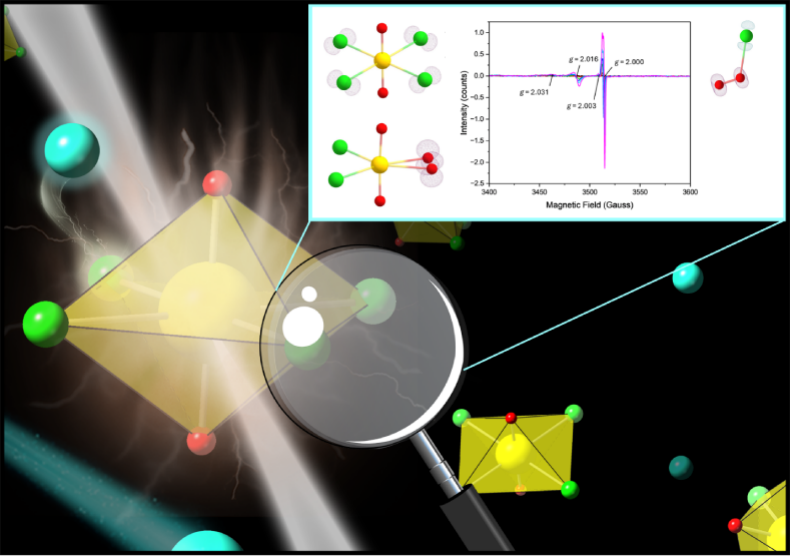

Nuclear materials, such as uranium-bearing solids, are exposed to high levels of ionizing radiation throughout the nuclear fuel cycle; thus, it is important to develop a molecular-level understanding of how these materials behave and degrade in the presence of gamma (γ) irradiation. In the current study, three U(VI) tetrachloride complexes, M_2_[UO_2_Cl_4_]·*x*H_2_O (where M = K^+^, Rb^+^, or Cs^+^ and *x* = 0 or 2), and their respective chloride salts were exposed to 1–50 kGy of γ radiation using a ^60^Co source. Irradiated materials were evaluated by using electron paramagnetic resonance (EPR) and Raman spectroscopy and were further explored by using density functional theory (DFT) methods. EPR spectra of the irradiated materials suggest the formation of a Cl-based radical for both the alkali salts and the uranyl tetrachloride compounds, and DFT calculations provide evidence that the Cl_2_^–•^ radical is formed within these materials. The presence of water in the K^+^ and Rb^+^ compounds leads to additional spectroscopic signatures that could be traced back to water radiolysis and the formation of peroxide and superoxide species. DFT results support the formation of HO_2_^•^ in the lattice and potentially the formation of a [UO_2_Cl_3_(O_2_)]^3–^ species, highlighting the impact of water within the hydrated material to alter U(VI) speciation by radiolysis.

## Abstract 2

Uranyl tetrachloride solid-state materials are model systems to explore their spectroscopic signatures upon ionizing radiation exposure, aiding in developing an atomistic understanding of the actinide-center contribution with radiolysis species.

## Introduction

Actinide solids are ubiquitous in light-water nuclear power generation, and the presence of fissile isotopes and fission/daughter products can lead to high levels of ionizing radiation that alter the chemistry and physics within the system.^1,2^ The most common types of ionizing radiation (α, β, and γ) in these systems can perturb the chemical environment by forming defects in the crystalline lattice, breaking chemical bonds, and forming reactive species (i.e., radicals).^3,4^ Exposure to radiation can also change the valence state of the actinide cation and alter the complexation environment, which can further impact material stability, solubility, and phase behavior.^5−7^ As such, it is important to understand the effects of ionizing radiation on actinide coordination complexes to comprehend the complex chemistry present within the extreme environment of high radiation fields.

Most of the previous work regarding the impact of ionizing radiation on actinide-bearing solids has focused on the structural stability and metal redox behavior in the presence of γ radiation.^8−11^ After irradiation, X-ray diffraction has been the main tool to evaluate material stability, where decreases in crystallinity were monitored by lower intensities and increased widths of the diffraction peaks.^12,13^ In addition, redox changes have been noted after irradiation of actinide-bearing solids,^14^ and the presence of vacancies and other defect sites in oxide materials has also been identified using spectroscopic techniques.^9,10^ While these studies provide a foundation for understanding the effects of ionizing radiation on actinide solids, additional efforts are needed to provide atomistic insights on the behavior of the metal cation and the overall impacts of radiation on a wider range of chemical constituents found in these materials.

Our prior work focused on the effects of γ radiation on solid-state U(VI) trinitrates, characterizing the radicals formed upon subsequent radiation doses.^15^ From this study, we were able to observe the formation of nitrate radicals using electron paramagnetic resonance (EPR) spectroscopy and identify changes in the U(VI) coordination environment from density functional theory (DFT) calculations. To expand on these efforts, we have chosen a second U(VI) model system based on the uranyl tetrachloride complex, [UO_2_Cl_4_]^2–^. This coordination complex was chosen to provide additional understanding of how variations in the equatorial ligand are impacted by radiation. In addition, the [UO_2_Cl_4_]^2–^ is a well-studied molecular building unit for coordination compounds and hybrid materials, and it is important in Cl-rich environments, including brines and ionic liquids.^16−19^ Herein, we report the effects of γ radiation on three solid-state U(VI)-containing systems, M_2_[UO_2_Cl_4_]·*x*H_2_O, where M = K^+^, Rb^+^, or Cs^+^ and *x* = 0 or 2, and their respective salts (i.e., KCl, RbCl, and CsCl), utilizing EPR and Raman spectroscopy. DFT calculations were also utilized to provide additional insights into the irradiation process.

## Experimental Section

### Synthesis of Uranyl Tetrachloride Compounds

*Caution: Uranyl acetate dihydrate ((UO_2_)(C_2_H_3_O_2_)_2_·2H_2_O) used in this study contains ^238^U. Standard precautions and licensing for handling radioactive substances should be followed by trained personnel.*

All reagents were used as received. Crystalline materials were synthesized by dissolving 1.2 g of uranyl acetate dihydrate (International Bioanalytical Industries, 98–102%) into 5.00 mL of 7 M HCl in a 20 mL scintillation vial. Two equimolar quantities of alkali metal chloride (KCl, RbCl, or CsCl) were added to the solution and mixed vigorously at 60 °C for 1 h until the formation of a transparent yellow solution. Blocky, fluorescent yellow crystals formed at the bottom of the vial through slow evaporation of the solution for several days. Samples were then allowed to evaporate to complete dryness. Final yields of crystallizations were 80–92% based on uranium . Attempts to use LiCl and NaCl did not result in the desired product.

### Powder X-Ray Diffraction

Crystalline U(VI) tetrachloro product and initial chloride salts (i.e., KCl, CsCl, and RbCl) were ground for 5 min to form a fine powder using an agate mortar and pestle. The solids were deposited onto a silica zero-background plate to form a thin polycrystalline layer. Samples were analyzed on a Bruker D-5000 powder X-ray diffractometer (Cu Kα = 1.54 Å) equipped with a LynxEye solid-state detector to determine the purity of the sample. Scans were performed from 5 to 60° 2θ with a step size of 0.02° 2θ and a count time of 0.5 s/step. Experimental patterns were collected to confirm bulk sample purity before and after irradiation (Figures S1–S3).

### Raman Spectroscopy

Raman spectra of the U(VI) product were collected on a Renishaw inVia Raman microscope equipped with a 785 nm laser and a 1200 mm grating, with a maximum operating power of 200 mW (laser power at 100%). Samples within the sealed EPR tubes were placed under the confocal microscope on the instrument, and the image was focused at 5× magnification to ensure alignment of the optics. Incremental scans were used to evaluate the optimal laser power and integration time for each sample by evaluating signal-to-noise ratios and the integrity of the material. The pinhole aperture, and thus the laser width, was 3 μm. An accumulation of 3 scans was performed on each sample, and an extended scan was collected from 2000 to 200 cm^–1^. To accurately process the Raman signals, the background was subtracted, and multiple peaks were fit using the peak analysis protocol with the pseudovoigt1 function the OriginPro 9.60 (OriginLab, Northampton, MA) 64-bit software. All the fitting parameters converged and correct fits were evaluated using R^2^ and χ^2^ results.

### X-Band EPR Spectroscopy

EPR spectra for the solid samples were collected on a Bruker EMX EPR spectrometer. Data were collected at room temperature using the Bruker Xenon software. The magnetic field was centered at 3500.00 G with a sweep width of 500.00 G, a 60.0 s sweep time, and a sample *g*-factor of 2.000. The magnetic frequency remained near 9.84 MHz, and the microwave attenuation and power were set to 20 dB and 2.00 mW, respectively. Modulation amplitude and frequency were also set at 1.000 G and 100.00 kHz, respectively. Five scans were collected per sample and were averaged within the software. Spectra were plotted in the OriginPro 9.60 (OriginLab, Northampton, MA) 64-bit software.

### Identification of Trace Metal Impurities

High-Resolution Inductively Coupled Plasma Mass spectrometry (HR-ICP-MS) was used to determine trace metal impurities within the Cs_2_[UO_2_Cl_4_] material evaluated in this study. Cs_2_[UO_2_Cl_4_] was dissolved in 3% HNO_3_ to achieve 10 ppb based on U. Samples were collected in triplicate on a Thermo Element XR High-Resolution ICP-MS and linear calibration curves were created using commercial ICP standards (Inorganic Ventures) diluted with Millipore water (18.2 Ω) in 3% HNO_3_.

### γ-Irradiation

*Caution: ^60^Co emits γ radiation. Irradiation experiments were carried out by trained personnel in a licensed research facility.*

Roughly 0.1130 g of finely ground polycrystalline samples were loaded into high-quality Suprasil EPR tubes. The EPR tubes were evacuated under vacuum for 20 minutes and then flame-sealed to ensure the systems remained air-free. The samples were placed in a Shepherd 109–68R Irradiator (^60^Co; *t*_1/2_ = 5.27 years, *E*_γ1_ = 1.17 MeV, and *E*_γ2_ = 1.33 MeV) and irradiated at room temperature at intervals to achieve total doses of 1, 5, 10, 20, and 50 kGy. The dose rate was determined using Fricke dosimetry, with an account for natural decay over the course of the experiments.^20^ EPR spectra of the material after each irradiation dose were performed as described for the preirradiated samples, and PXRD, EPR, and Raman spectroscopy were collected on all samples after the final radiation dose.

### Density Functional Theory Calculations

The Gaussian 16^21^ software package was used to perform all geometry optimizations of molecular species. The B3LYP (Becke, 3-parameter, Lee–Yang–Parr)^22,23^ hybrid functional was used to model exchange-correlation effects and van der Waals dispersion correction methods. DFT-D3 with the Becke–Johnson damping term was used.^24^ A polarized triple-ζ (def2-TZVP)^25^ basis set was utilized to represent the O, H, and Cl atoms, while the SDD effective core potential and corresponding valence basis sets were used to represent U atoms.^26,27^ Calculated vibrational frequencies from these geometric optimizations were calculated and noted to ensure that structures were optimized to a true minimum with no imaginary frequencies. When calculating reaction energies, the conductor-like polarizable continuum solvation model (CPCM)^28,29^ with water as the solvent was used during both geometry optimization and final single-point calculations that yield thermodynamic parameters. The inclusion of solvation in energetic calculations is justified by the assumption that these reactions may occur under aqueous conditions due to surface-bound water on the crystals.

The calculation of the *g*-factor for the optimized molecular structures was performed by ORCA 6.0.1^30^ using the B3LYP hybrid functional.^22,23^ For these calculations, the realistic effects are included by the zeroth-order regular relativistic approximation (ZORA)^31,32^ in combination with ZORA-recontracted^33^ versions of the def2 basis sets.^34,35^ The H, O, and Cl atoms are represented by the ZORA-def2-TZVP basis set, while U atoms are represented by the SARC-ZORA-TZVP basis set together with SARC/J Coulomb-fitting auxiliary basis sets.^33−35^ Tight SCF convergence was used throughout the calculation of all EPR parameters. Visualization of spin densities was done using the Chemcraft program.^36^

## Results and Discussion

### Characteristics of As-Synthesized Materials

**Figure 1:**
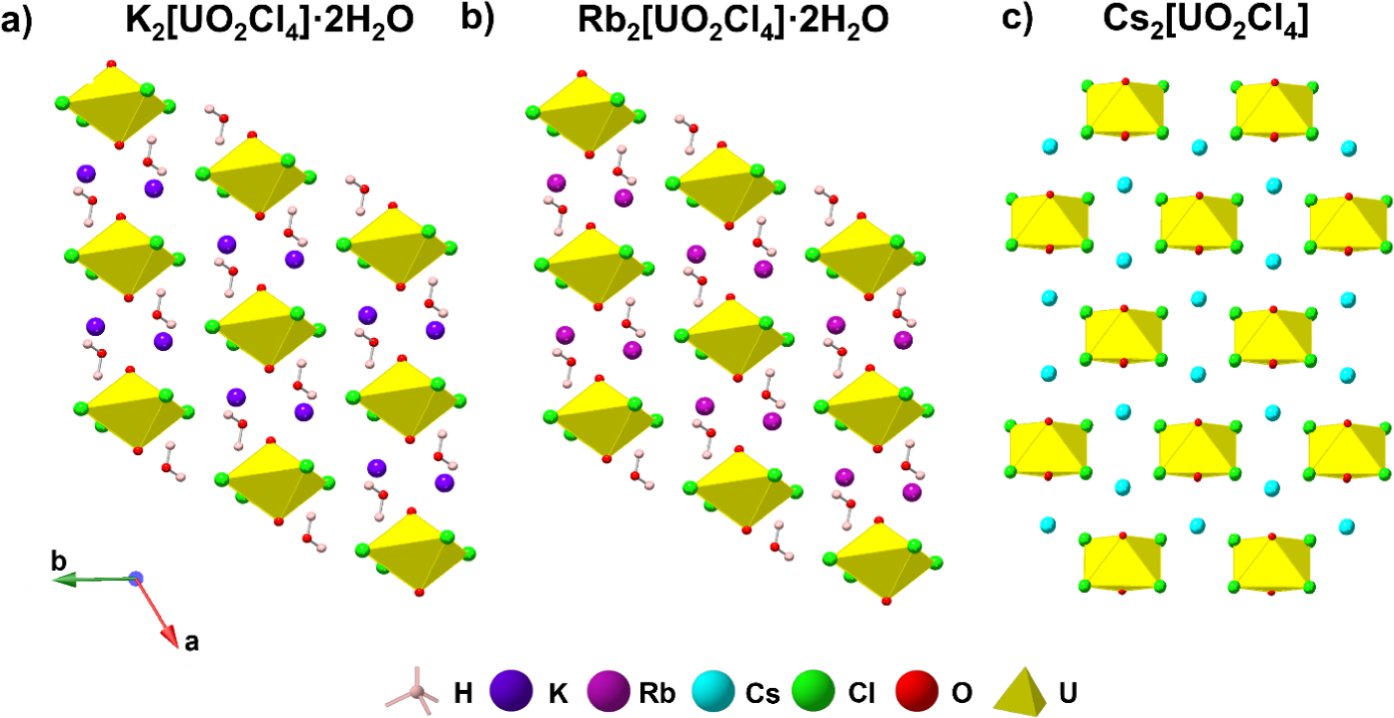
Crystal packing of the (a) K_2_[UO_2_Cl_4_]·2H_2_O, (b) Rb_2_[UO_2_Cl_4_]·2H_2_O, and (c) Cs_2_[UO_2_Cl_4_] solids^37^ analyzed in this study. H, K, Rb, Cs, Cl, O, and U are colored light pink, purple, dark pink, teal, green, red, and yellow, respectively.

Powder X-ray diffraction experiments confirmed the identity of the potassium, rubidium, and cesium uranyl tetrachloride phases (Figures S1–S3). Both K_2_[UO_2_Cl_4_]·2H_2_O and Rb_2_[UO_2_Cl_4_]·2H_2_O are isostructural to one another, where the central U(VI) cation strongly bonds to two oxygen atoms to form the nearly linear UO_2_^2+^ moiety and is further coordinated about the equatorial plane by four Cl^–^ anions, resulting in a square bipyramidal geometry (Figure 1a,b). Within these phases, the [UO_2_Cl_4_^2–^] coordination complex engages in additional electrostatic interactions with the alkali cations (K or Rb) in the second coordination sphere, with additional hydrogen bonding interactions occurring between interstitial water molecules located in the lattice and coordinated Cl^–^ anions. The same uranyl tetrachloride complex occurs in Cs_2_[UO_2_Cl_4_], but the overall crystal packing differs due to the anhydrous nature of the solid-state complex (Figure 1c).

**Figure 2:**
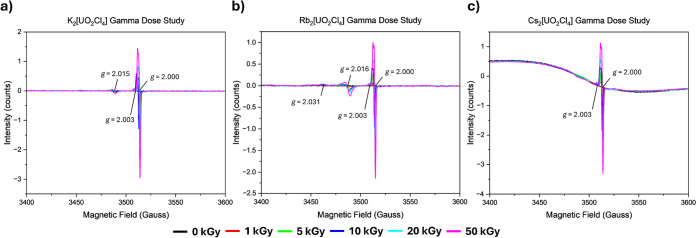
EPR spectra for γ radiation doses studies with associated *g*-factors of (a) K_2_[UO_2_Cl_4_]·2H_2_O, (b) Rb_2_[UO_2_Cl_4_]·2H_2_O, and (c) Cs_2_[UO_2_Cl_4_]. Doses are represented by different colors, as described in the legend provided.

The uranyl tetrachloride solids were analyzed by Raman and EPR spectroscopies before radiation exposure. The symmetric stretch of the uranyl unit is highly sensitive to changes in both the primary and secondary coordination spheres.^38^ In the primary coordination sphere, factors such as (i) an increase in the overall negative charge of the complex, (ii) an increase in the number of coordinated ligands, and (iii) substitution of weak electron-donating ligands with stronger ones (e.g., replacing chloride with peroxide) typically result in a red shift of the uranyl Raman band.^38^ Additionally, secondary interactions—including actinyl–hydrogen bonding, actinyl–cation interactions, and actinyl–actinyl contacts—can further contribute to red-shifting. Therefore, Raman spectroscopy serves as an effective tool for probing subtle changes in the uranyl coordination environment.^39−41^ Prior to irradiation, the Raman spectra for K_2_[UO_2_Cl_4_]·2H_2_O matches previous literature reports, where the uranyl symmetric stretch (ν_1_ UO_2_^2+^) appears at 836 cm^–1^ (Figure S4), and the ν_4_ and ν_5_ of the U–Cl bond appear at 266 and 229 cm^–1^, respectively. Additional features in the spectra include K–O stretching modes at 213 and 205 cm^–1^ and two weak and broad water libration modes that appear at 420 and 310 cm^–1^ associated with the interstitial water within the solid-state lattice. Similarly, the ν_1_ UO_2_^2+^ for Rb_2_[UO_2_Cl_4_]·2H_2_O occurs at 833 cm^–1^ (Figure S6), and the ν_4_ and ν_5_ of the U–Cl bond appear at 262 and 227 cm^–1^. The Rb–O stretching modes appear at 214 and 204 cm^–1^, but no significant water libration modes are observed in the Raman spectra for the Rb phase. Only three features are noted for Cs[UO_2_Cl_4_], including the ν_1_ UO_2_^2+^ at 829 cm^–1^ and ν_4_ of the U–Cl bond at 260 cm^–1^ and the Cs–O feature at 200 cm^–1^ (Figure S8). Both dihydrate systems, K_2_[UO_2_Cl_4_]·2H_2_O and Rb_2_[UO_2_Cl_4_]·2H_2_O, contained no features in the EPR spectra prior to irradiation (Figure 2a,b). However, Cs_2_[UO_2_Cl_4_] contains a large paramagnetic feature in its starting form (Figure 2c). This feature is due to the presence of a Mn^2+^ impurity in this sample, confirmed via HR-ICP-MS (Tables S1–S3) and further verified with EPR spectroscopy of MnCl_2_ (Figure S10). The presence of a Mn^2+^ contaminant is not unreasonable for this system, as it is a common metal impurity in the alkali metal chloride starting material.

### γ-Irradiation of Solid Materials

To properly assess the features observed in the uranyl tetrachloride system, their related alkali salts (KCl, RbCl, and CsCl) were first irradiated as controls. The most prominent features in the EPR spectra of all three chloride salts after exposure to γ radiation are an isotropic peak at *g* = 2.000, with an additional small peak at *g* = 2.003 (Figures S11–S13). We attribute the *g* = 2.000 peak to the formation of an F-center due to the irradiation of the quartz EPR tube; however, the identity of the *g* = 2.003 peak was not completely clear. Our initial assessment was that it was associated with a chlorine-based radical species.

When the uranyl tetrachloride phases are exposed to ionizing radiation, we observe the ingrowth of two spectroscopic signatures in all samples, including two sharp features overlaid at *g* = 2.000 and a small isotropic signature at *g* = 2.003 (Figure 2). The sharp feature at *g* = 2.000 is again associated with the F-center defects that appear upon irradiation of the quartz tube. The small isotropic feature occurs at *g* = 2.003 and is saturated by 5 kGy of radiation exposure. Irradiation of the K_2_[UO_2_Cl_4_]·2H_2_O and Rb_2_[UO_2_Cl_4_]·2H_2_O compounds results in additional features in the EPR spectra (Figure 2a,b). Within the spectra for Rb_2_[UO_2_Cl_4_]·2H_2_O, there are additional axial features present at *g*_||_ = 2.031 and *g*_*⊥*_ = 2.016 that increase with the dose. Interestingly, for K_2_[UO_2_Cl_4_]·2H_2_O, there is a *g*_⊥_ = 2.016 but no presence of a *g*_||_ = 2.031, even though this material is isostructural to Rb_2_[UO_2_Cl_4_]·2H_2_O. It is plausible this effect may be similar to reports by Lushchik *et al*. that examined excitonic and electron–hole mechanisms in alkali metal halides, where different mechanisms were observed between KCl and RbCl upon irradiation.^42^ These studies suggest Rb could increase the stability of the generated radical species upon irradiation,^42^ potentially increasing interactions between the superoxide species and the U(VI) center, perturbing the U(VI) primary coordination sphere, and altering the EPR signature to axial symmetry. Additionally, these signatures are not observed in the related alkali chloride salts, suggesting that either the presence of U(VI) or the water molecules in the lattice may result in the observed signatures.

The presence of these axial signatures suggests that radical interactions with 5*f* orbitals of the UO_2_^2+^ center influence the EPR signals observed in this study. Studies by Zimbrick *et al*. and Gunter *et al*. evaluated the presence of radicals formed in the irradiation of pure water and did not observe any axial spectroscopic signatures.^43,44^ In this case, the major radiolysis products were OH^•^ or HO_2_^•^, where the latter has a rhombic signature at *g*_z_*=* 2.035, *g*_y_ = 2.0075, and *g*_x_ = 2.004. Prior work by Scherrer *et al*. demonstrates that the axial signature is present when the O_2_^–•^ is stabilized by the U(VI) cation. Rhombohedral signatures have been reported by Kruse *et al*. for uranyl nitrate systems when the axial symmetry of the molecule is disrupted by changes in the bonding mode of the ligands^15,45^

**Figure 3:**
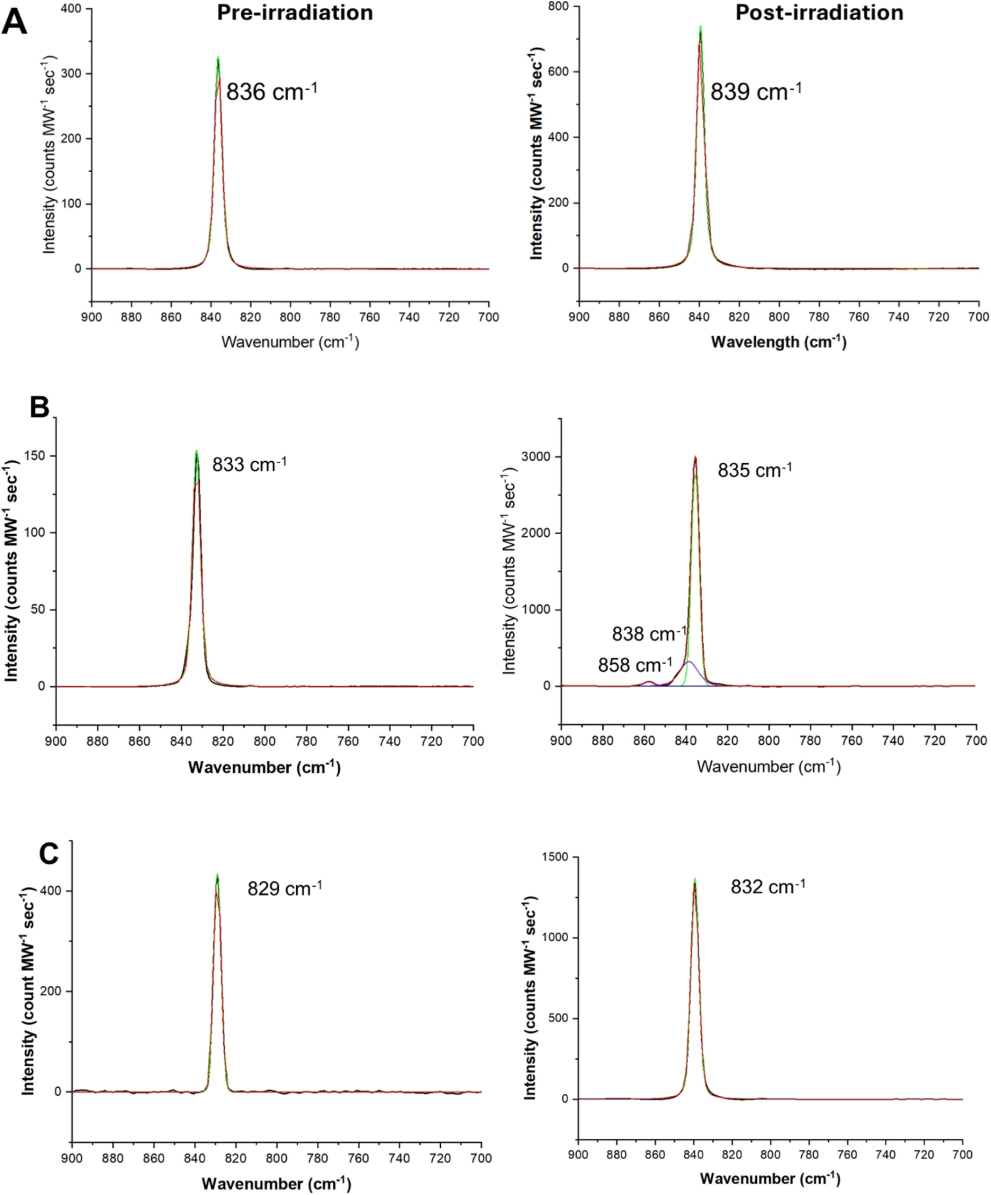
Fit Raman spectra within the region of interest for (Aa) K_2_[UO_2_Cl_4_]·2H_2_O, b(B) Rb_2_[UO_2_Cl_4_]·2H_2_O, and c(C) Cs_2_[UO_2_Cl_4_] before and after 50 kGy of γ radiation exposure.

To further assess changes to each material upon irradiation, Raman spectroscopy was used to determine the in-growth of additional features. Upon irradiation of K_2_[UO_2_Cl_4_]·2H_2_O, a slight blue shift in the ν_1_ UO_2_^2+^ mode to 839 cm^–1^ was noted in the spectra; however, this change in wavenumber is within the error of the system (Figure 3A).^46^ Similarly, the ν_1_ UO_2_^2+^ stretching vibration in Rb_2_[UO_2_Cl_4_]·2H_2_O and Cs_2_[UO_2_Cl_4_] also blue-shifts by 2 and 3 cm^–1^, respectively, which again we assume to be within error (Figure 3B,C).^46^ However, additional spectral features appear in the Raman spectra of Rb_2_[UO_2_Cl_4_]·2H_2_O after 50 kGy of γ-irradiation (Figure 3B). Within our typical spectral window of interest, there are two peaks, where the first is a weak band located at 858 cm^–1^ and the second occurs at 841 cm^–1^.^46^ The identity of these peaks is not entirely clear, as there can be multiple interpretations of the bands present. For example, the band at 858 cm^–1^ agrees well with the ν_1_ symmetric stretch for uranyl within the [UO_2_Cl_4_(H_2_O)] complex (previously reported at 859 cm^–1^),^46^ but is also in the region for the hydrated peroxide feature within Rb_2_O_2_ (855 cm^–1^).^47^ Thus, we turned to computational analysis to provide additional insights into the system.

### DFT Guided Analysis of the Irradiated Materials

**Figure 4:**
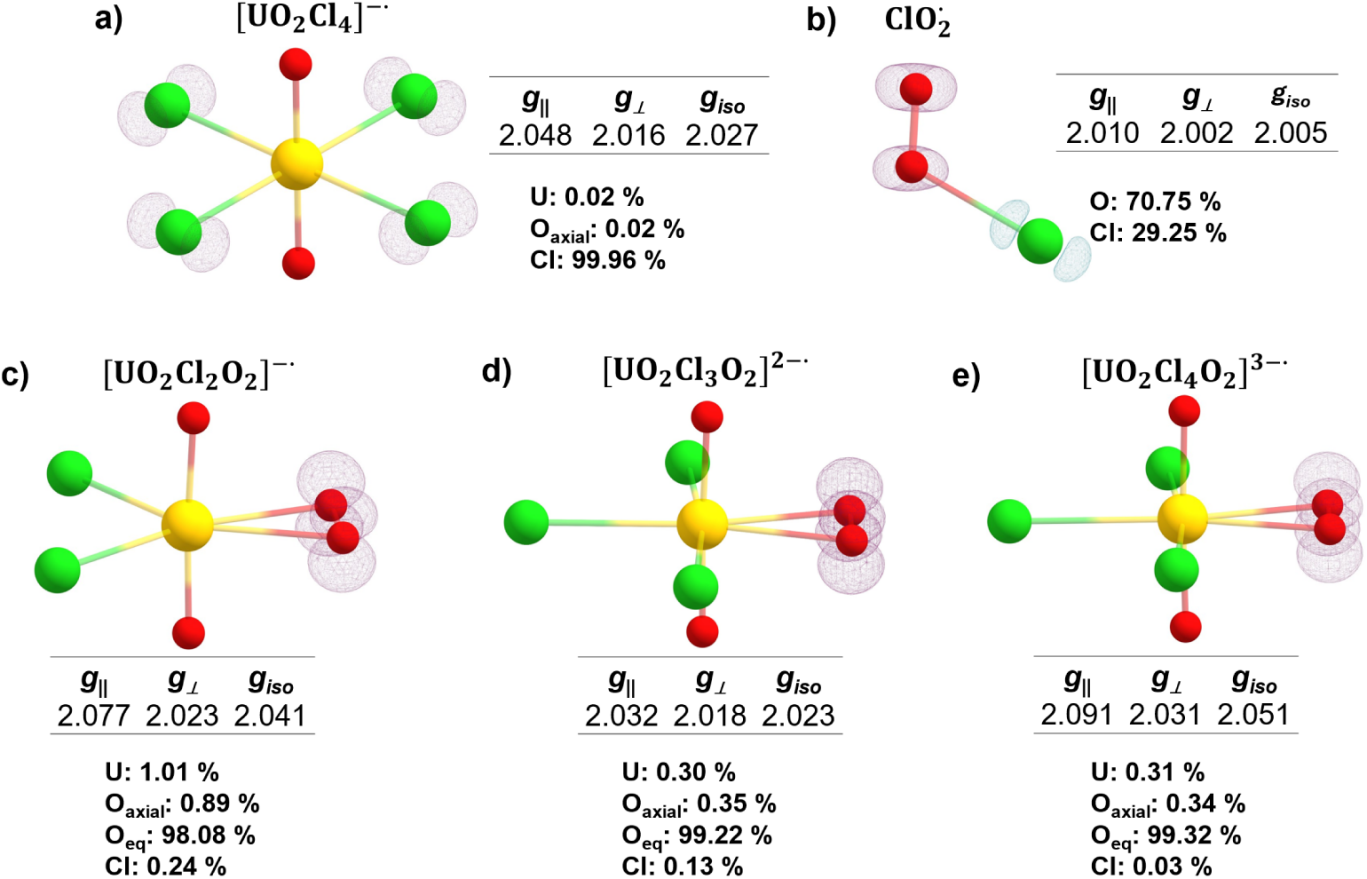
Calculated *g*-factors, spin densities, and atomic distribution of spin density of (a) [UO_2_Cl_4_]^−•^, (b) ClO_2_^•^, (c) [UO_2_Cl_2_(O_2_)]^−•^, (d) [UO_2_Cl_3_(O_2_)]^2–•^, and (e) [UO_2_Cl_3_(O_2_)]^3–•^. The isosurfaces of the spin densities were generated with a contour value of 0.012. The atomic distribution of spin density of (a) indicates the radical is primarily on Cl, while spin density of (c-–e) shows that the radical is primarily on equatorial oxygen, resembling a superoxide radical.

DFT calculations for the optimized [UO_2_Cl_4_]^−•^ species yielded *g*_||_ = 2.048 and *g*_⊥_ = 2.016 (Figure 4). The calculated *g*_⊥_ values align well with the experimentally observed feature at 2.014–2.016 in K_2_[UO_2_Cl_4_]·2H_2_O and Rb_2_[UO_2_Cl_4_]·2H_2_O but are absent in Cs_2_[UO_2_Cl_4_]. Here, the calculated spin density(ρ_s_(**r**)), defined as the difference between α and β electron densities [ρ_s_(**r**) = ρ_α_(**r**) – ρ_β_(**r**)], localizes on chlorine, indicating a chlorine-centered unpaired electron (Figure 4). One could argue that the lack of this signature could be caused by overlap with the broad *g* = 2.015 signal from Mn impurities, which obscures the sharp, narrow feature expected for a chlorine-centered radical.^48^ However, the *g* = 2.048 feature corresponding to *g*_||_ is absent in all recorded EPR spectra. The absence could be attributed to the relatively low intensity of *g*_||_ compared to that of *g*_⊥_, but typically, we do observe the anisotropic signatures when free radicals are bound to the uranyl cation. Therefore, we determined that the results from these initial DFT calculations did not conclusively confirm the formation of [UO_2_Cl_4_]^−•^. We therefore broadened our computational approach to examine a wider range of paramagnetic species potentially formed under ionizing radiation, including radicals derived from chloride and water radiolysis.

K_2_[UO_2_Cl_4_]·2H_2_O and Rb_2_[UO_2_Cl_4_]·2H_2_O compounds contain crystalline water, and all three crystal phases may also exhibit adsorbed water due to their hygroscopic nature. To understand potential radiolytic products formed under ionizing radiation, we considered the generation of radical species from water and chloride and calculated their *g*-factors by using our DFT methods. Radiolysis in chloride-rich aqueous media is known to yield both diamagnetic species (e.g., H_2_O_2_ and H_2_) and paramagnetic species (O_2_, HO_2_^•^, O_2_^–•^, H_2_O^+•^, ^•^OH, H^•^, Cl^•^, and ClO_2_^•^) as reported in prior studies.^49−53^ These previously reported experimental values and our calculated *g*-factors are summarized in Table 1

**Table 1:** Calculated and Reported Experimental *g*-Factors of Radiolytic Products of Water- and Chlorine-Based Radicals without Spin Traps

	**Calculated**			**Experimentally reported**		
**Radical**	***g*_||_**	***g*_⊥_**	***g*_iso_**	***g*_||_**	***g*_⊥_**	***g*_iso_**
H^•^	-	-	2.002	-	-	2.002, *A*_H_ = 500 G^54,55^
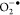	2.175	2.006	2.062	2.034–2.168^56−58^	2.005 −2.075^56−58^	2.015 −2.106^56−58^
H_2_O^+•^	2.015	2.004	2.008	-	-	-
HO^•^	2.067	2.005	2.025	-	-	-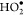	2.033	2.005	2.014	2.039^59^	2.004^59^	2.016^59^
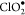	2.010	2.002	2.005	2.013^60^	2.000^60^	2.004^60^
Cl^•^	2.002	2.020	2.014	2.002^48,61^	2.025^48,61^	2.017^48,61^
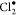	2.051	2.002	2.034	-	-	2.007^62^

An isotropic *g*_iso_ signal would be observed in free water or adsorbed due to the free rotational mobility of molecules. Therefore, we initially considered solvated molecules as plausible species present in the hydrated materials. Among the paramagnetic species evaluated, ClO_2_^•^ was anticipated to be a strong candidate due to its calculated *g*_iso_ value closely matching the experimentally observed isotropic feature at *g*_iso_ = 2.003. While H^•^ also has a similar *g*_iso_ value, its characteristic hyperfine splitting (*A*_H_ = 500 G) makes its presence unlikely. The calculated *g*_iso_ for H^•^ likely results from the averaging of its typical doublet, centering around *g*_iso_ = 2.002, as previously noted by Stoll *et al*.^55^ This reinforces the conclusion that ClO_2_^•^ is the primary contributor to the observed signals. In contrast, for crystalline water, where rotational freedom is hindered, the comparison of *g*_||_ and *g*_⊥_ factors becomes more appropriate. The calculated *g*_||_ and *g*_⊥_ values for H_2_O^+•^ closely match the experimentally observed *g*-factors for K_2_[UO_2_Cl_4_]·2H_2_O and Rb_2_[UO_2_Cl_4_]·2H_2_O, but this species is highly reactive and not expected to survive.

With the presence of water in the lattice, other radiolysis products were also considered, including reactive oxygen species with EPR signatures. Uranium peroxide phases, such as studtite ([(UO_2_)O_2_(H_2_O)_2_]·2H_2_O), have been reported to occur from γ–irradiation of uranium oxides, where H_2_O_2_ is generated via radiolysis of water and readily binds to the uranyl.^63−67^ In our previous work on uranyl peroxide complexes, we observed the ingrowth of EPR-active superoxide species over time. In the case of studtite, superoxide exhibited features at *g*_||_ = 2.035–2.039 and *g*_⊥_ = 2.014–2.015^45^ and similar superoxide uranyl triperoxide features were observed at *g*_||_ = 2.050 and *g*_⊥_ = 2.017.^68^ EPR features of studtite are similar to the experimental features detected in irradiated Rb_2_[UO_2_Cl_4_]·2H_2_O, which displayed *g*_||_ = 2.031–2.034 and *g*_⊥_ = 2.014–2.016. To investigate the feasibility of the reaction further, we computed the thermodynamic stability via enthalpy change (Δ*H*) and free energy change (Δ*G*) (Table 2), and *g*-factors (Figure 4c–e) of potential uranyl chloroperoxide and superoxide complexes. From these calculations, we determined that the formation of [UO_2_Cl_2_(O_2_)]^2–^, [UO_2_Cl_3_(O_2_)]^3–^, and [UO_2_Cl_3_(O_2_)]^2–•^ complexes was thermodynamically favorable. In addition, the calculated *g*-factors of [UO_2_Cl_3_(O_2_)]^2–•^ (*g*_||_ = 2.032 and *g*_⊥_ = 2.018) fall within the range of the observed EPR features in postirradiated Rb_2_[UO_2_Cl_4_]·2H_2_O. This matched with previous observations by Li *et al*., where uranyl peroxo-chloro complexes form in the presence of peroxide and chloride anions.^69^ The localization of spin density primarily on the superoxide in the [UO_2_Cl_3_(O_2_)]^2–•^ species can be rationalized by examining the molecular orbitals of the parent [UO_2_Cl_3_(O_2_)]^3–^ complex. The HOMO and HOMO–1 are predominantly composed of peroxide  and  orbitals, with minimal contribution from uranyl orbitals (Figure S32). Upon one-electron oxidation to form [UO_2_Cl_3_(O_2_)]^2–•^, the electron is removed from the HOMO, leaving the unpaired electron in the  orbital of the peroxide. As a result, the spin density remains primarily localized on the superoxide with limited involvement of the uranyl center. It is important to note that this study considers only the thermodynamic stability of the products without accounting for kinetic effects. However, since the postirradiation experiments were not monitored in situ and occur over time scales of hours, thermodynamic control is likely more relevant than kinetic factors.

**Table 2:** Calculated Δ*H* and Δ*G* for the Formation of Uranyl Chloro Peroxide and Uranyl Chloro Superoxide Complexes

**Reactions with peroxide**	**Δ*H***(kJ/mol)	**Δ*G***(kJ/mol)
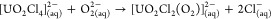	–84.65	–68.95
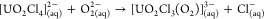	–89.72	–77.00
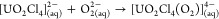	a	a

**Reactions with superoxide**	**ΔH**(kJ/mol)	**ΔG**(kJ/mol)
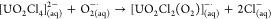	–8.05	8.75
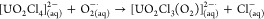	–21.38	–9.15
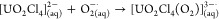	–4.55	3.5

a ^∗^The product of this reaction is does not remain intact during the optimization, thus could not be optimized without constraints.

Taking all of the data together, we can provide an overall assessment of the irradiation of the uranyl tetrachloro systems. The *g* = 2.003 feature is observed in the alkali chloride salts, as well as in the hydrated and dehydrated uranyl tetrachloro systems. This observation suggests that it is due to the presence of chloride in the sample and not the U(VI) cation. We can eliminate most of the possible radicals except the ClO_2_^•^ (*g*_||_ = 2.010 and *g*_⊥_ = 2.002), Cl_2_^•–^ (*g*_||_ = 2.051 and *g*_⊥_ = 2.002), and Cl^•^ (*g*_||_ = 2.002 and *g*_⊥_ = 2.020) species. For this to be the correct assignment, one has to assume that the intensity of the g_||_ for ClO_2_^•^ or Cl_2_^•–^ is too low to be detected, or that we are observing the *g*_iso_ value. Prior studies on the irradiation of chloride salts by Ramos-Ballesteros *et al*. noted a feature at 2.007 for solid KCl exposed to γ radiation, which was assigned to Cl_2_^•–^. Overall, γ-irradiation of the uranyl tetrachloro system leads to the formation of a chloride-based radical that appears to not be coordinated with the U(VI) center. However, the exact identity of the radical species cannot be conclusively determined based on the current data.

The features at *g* = 2.016 and 2.032 are assigned to the hydrated tetrachloro phases, which is likely due to the formation of multiple superoxide species. Within the Rb_2_[UO_2_Cl_4_]·2H_2_O compound, the calculated *g*-factor and the overall energetics suggest that the [UO_2_Cl_3_(O_2_)]^2–•^ complex forms under irradiation. However, the formation of both the free HO_2_^•^ and the O_2_^–•^ species is also a possibility, as they have calculated *g*-factors that are near experimental values. In addition, the Raman features at 858 and 838 cm^–1^ are similar to those reported by Eysel and Thym for the O_2_^2–^ stretch of the “hydrated” species (i.e., at 855 and 830 cm^–1^).^47^ Therefore, we can determine that radiolysis of the lattice water molecules likely results in the formation of superoxide and peroxide species that can coordinate to the alkali cations or the uranyl cation. In addition, the release of a bound Cl^–^ anion from [UO_2_Cl_4_]^2–^ would support the formation of the chloride radical species and the [UO_2_Cl_3_(O_2_)]^2–•^ complex.

It is also interesting to note that the presence of different countercations affects the EPR signatures, especially with regard to the dihydrate systems. It is plausible that this effect may be similar to reports by Lushchik *et al*. that examined excitonic and electron–hole mechanisms in alkali metal halides, where different mechanisms were observed between KCl and RbCl upon irradiation.^42^ This effect is attributed to the formation of dihalide radical (X_2_^•^) holes within the lattice, where KCl was observed to have larger interdefect distances that prevent the stabilization of the reactive species formed, whereas RbCl exhibited the opposite. Thus, within each M_2_[UO_2_Cl_4_]·2H_2_O (where M = K or Rb), the same effect may take place, where the formation of a longer-lived superoxide is prolonged due to interactions with neighboring Rb^+^ such that coordination can take place with the uranyl cation, whereas K^+^ does not. However, further studies are necessary to verify the exact mechanism in action. The DFT calculation does not include the secondary coordination sphere into account, and the counterion and hydrogen bonding may have a significant influence on the thermodynamic and spectral properties of the investigated molecules. Thus, further studies are necessary to investigate the effect of the secondary coordination sphere on uranyl-coordinated radicals.

## Conclusion

In this present work, we investigated the effects of radiation on M_2_[UO_2_Cl_4_], where M = K^+^, Rb^+^, and Cs^+^, solid-state materials, to determine the identity of the resulting radical species. The combination of EPR spectroscopy, Raman spectroscopy, and computational calculations enabled the detailed characterization of reactive species formed upon irradiation, with good agreement between experimental and theoretical results. Notably, there is compelling evidence for the formation of Cl^–^ based radicals and the formation of superoxide radicals in the form of HO_2_^•^ or O_2_^–•^ and [UO_2_Cl_3_(O_2_)]^2–•^ radicals within hydrated materials. This result highlights the importance of water radiolysis for hydrated materials and how these radiolysis products can impact the overall speciation of the U(VI) and the resulting degradation pathway.

Additional experimental and computational efforts are needed to fully explore the impacts of radiation on actinide solids. The formation of free radicals is highly dependent on the nature of the ligands in these systems, and these species can impact the behavior and coordination environment around the actinide metal cation. In addition, the stability of the specific free radicals in solid-state materials may be related to the chemical environment, which, in this particular study, was the alkali metal cations; however, these effects are not well understood within solid-state systems. Lastly, the presence of water in the crystalline lattice will induce the formation of reactive oxygen-based radicals that may trigger a cascade reaction, altering the nature of the overall compound. Understanding these effects at an atomistic level will enhance our understanding of actinides in extreme environments, such as ionizing radiation fields, and provide opportunities to develop new strategies for the safe containment of nuclear materials.

## Supporting Information Available

Powder diffractograms and Raman spectra of M_2_[UO_2_Cl_4_]]·*x*(H_2_O) (M = K, Rb, or Cs and *x* = 0 or 2), ICP-MS analysis on Mn(II) impurity, EPR spectra of dose studies done on uranyl tetrachloride complexes and alkaline metal chlorides, and DFT optimized bond lengths and geometries (PDF)
