# Supplementary material for: Radiation Effects on Uranyl Tetrachloro Coordination Compounds: Impact of Lattice Water
Source: Inorg Chem. 2025 May 7;64(19):9652–61. doi: 10.1021/acs.inorgchem.5c00693 (PMC12093375; doi:10.1021/acs.inorgchem.5c00693)
Supplement: Supplementary file 1 — ic5c00693_si_001.pdf [file ic5c00693_si_001.pdf]

Radiation Effects on Uranyl Tetrachloro Coordination Compounds: Impacts of Lattice Water

Harindu Rajapaksha[a,†], Samantha J. Kruse[a,b,†], Jay A. LaVerne[b], Sara E. Mason[a,c]*, Tori Z. Forbes[a]*

[a]*Department of Chemistry, University of Iowa, Iowa City, IA, USA, 52242*

[b]*Department of Physics and Astronomy, University of Notre Dame, Notre Dame, IN, USA, 46556*

[c]*Center for Functional Nanomaterials, Brookhaven National Laboratory, Upton, NY, USA, 11973*

†These authors contributed equally to this work and share first authorship

*Corresponding Author: Tori Z. Forbes Email: tori-forbes@uiowa.edu

Supporting Information

Table of Contents

PXRD

Diffractograms of $M_2[UO_2Cl_4]\,]\cdot x(H_2O)$ (M = K, Rb, or Cs and x = 0 or 2)……………......….S2

Raman Spectroscopy

$K_2[UO_2Cl_4]\cdot 2(H_2O)$……………………………………………………………………..S5

$Rb_2[UO_2Cl_4]\cdot 2(H_2O)$………………………………………………………………………..S6

$Cs_2[UO_2Cl_4]$……………………………………………………………………………………S7

Mn(II) Impurity

ICP-MS ……………………………………………………………………………… S8

EPR………………………………………………………………………………………………S10

DFT

DFT optimized bond lengths of the uranyl complexes……………………………………...S17

DFT optimized geometries………………………………………………………………...…S21

References………………………………………………………………………………………S24

Powder X-ray Diffraction (PXRD)

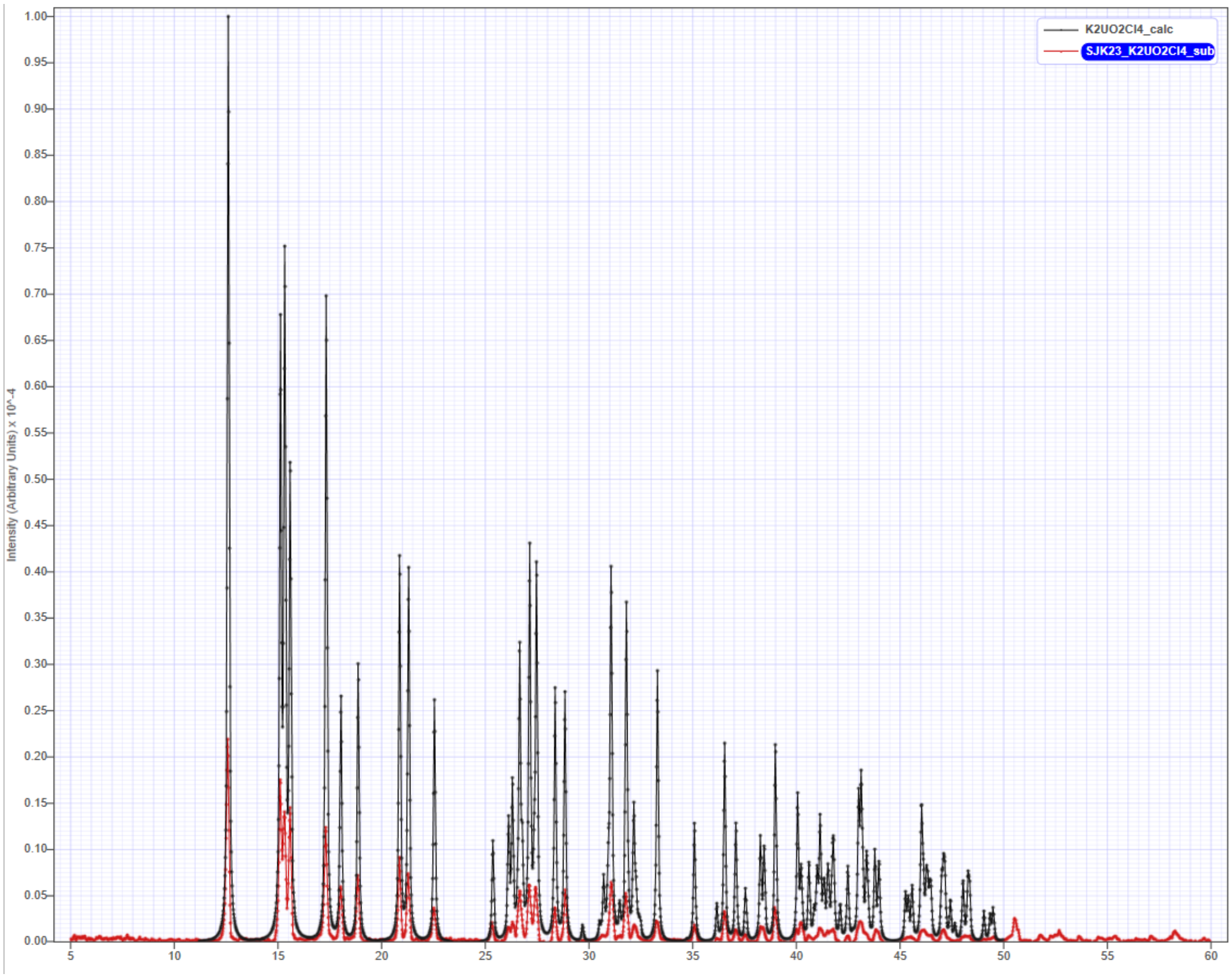

Figure S1. Overlay of $K_2[UO_2Cl_4]\cdot 2H_2O$ of the calculated (black) and experimental (red) powder patterns.

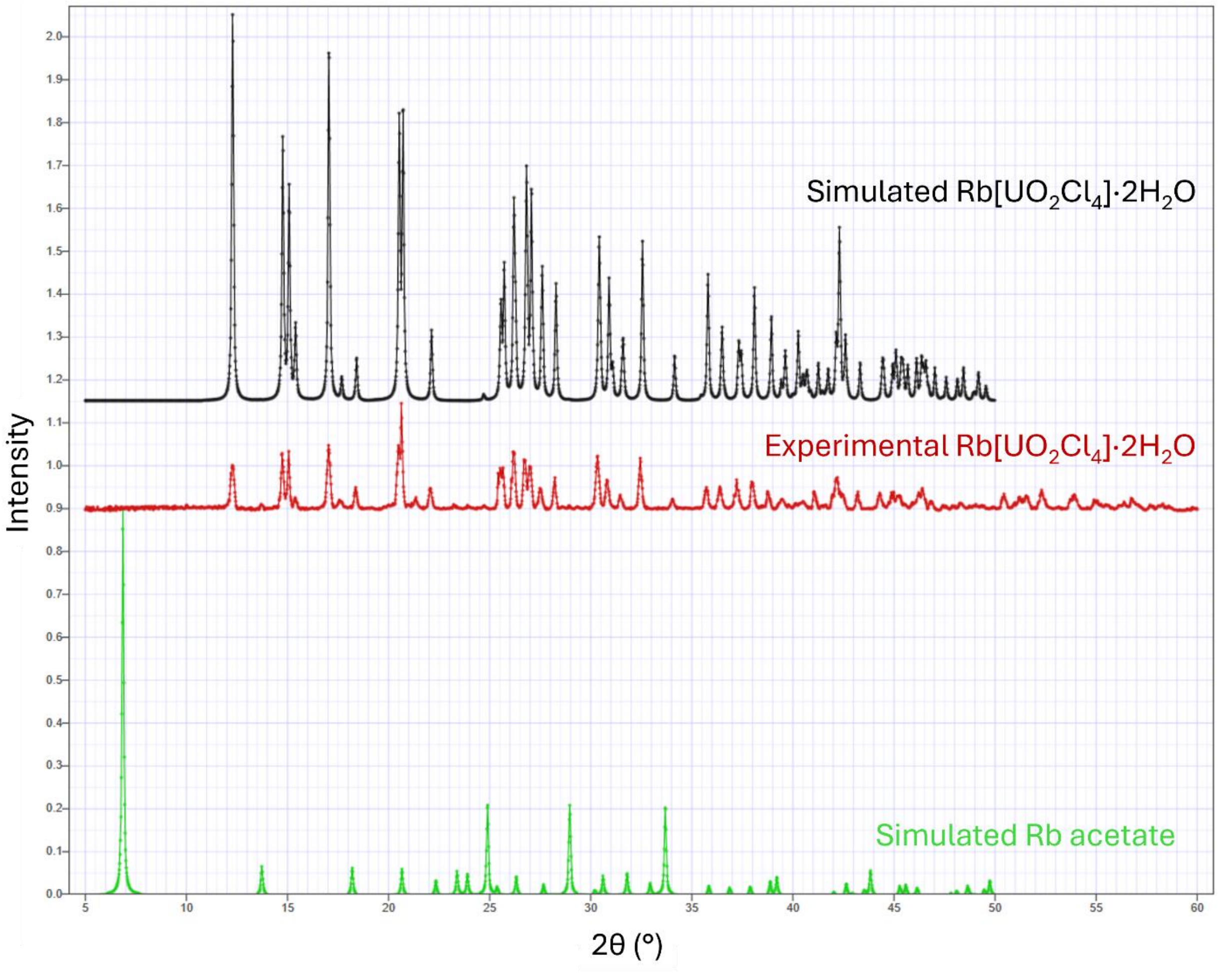

Figure S2. Powder pattern of $Rb_2[UO_2Cl_4]\cdot 2H_2O$ simulated (black), experimental (red), and simulated Rb acetate (green).

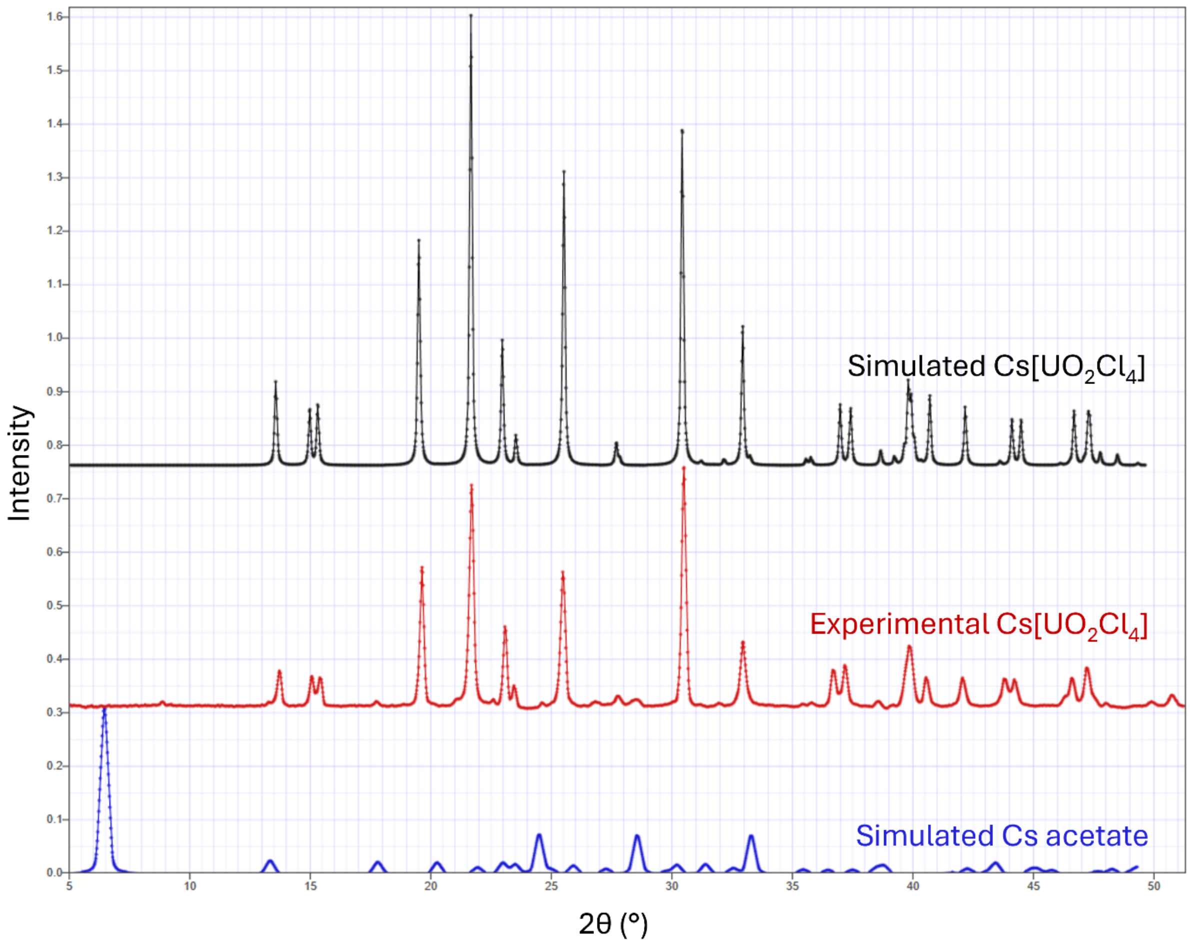

Figure S3. Powder pattern of $Cs_2[UO_2Cl_4]$ simulated (black), experimental (red), and simulated Cs acetate (blue).

Raman Spectroscopy

Raman Spectra of $K_2[UO_2Cl_4]$ before and after γ radiation

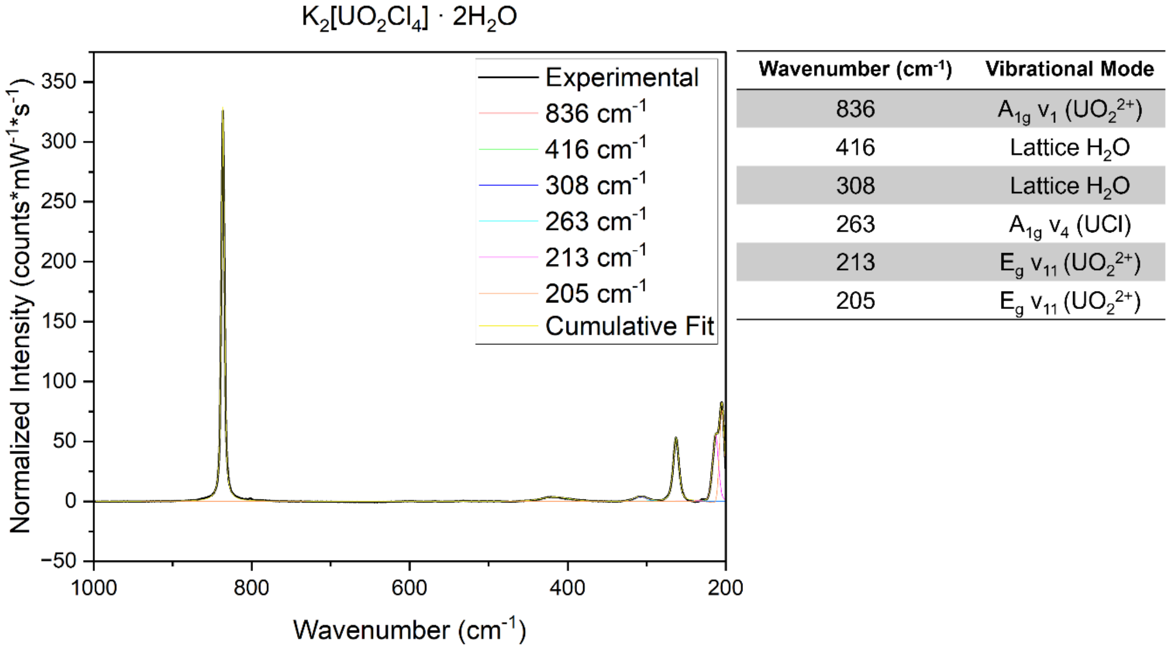

Wavenumber (cm^{-1})	Vibrational Mode
836	A_{1g} v_1 (UO_2^{2+})
416	Lattice H_2O
308	Lattice H_2O
263	A_{1g} v_4 (UCl)
213	E_g v_{11} (UO_2^{2+})
205	E_g v_{11} (UO_2^{2+})

Figure S4. Fitted Raman spectrum of the $K_2[UO_2Cl_4]$ solid pre-irradiation.[2, 3]

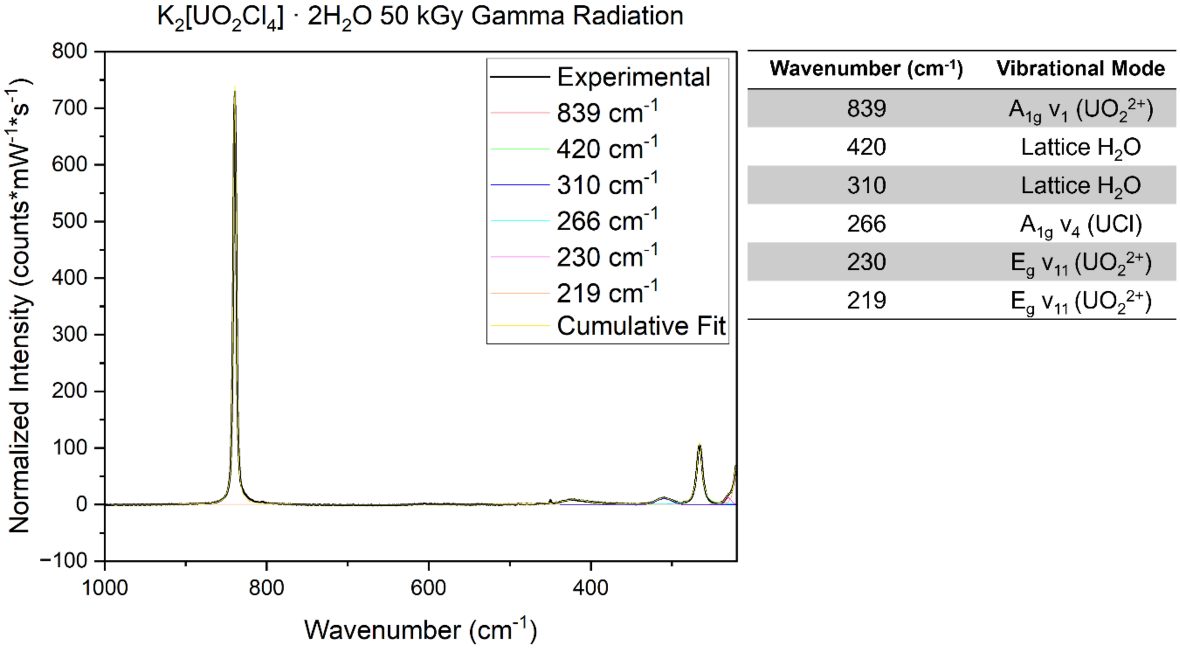

Wavenumber (cm^{-1})	Vibrational Mode
839	A_{1g} v_1 (UO_2^{2+})
420	Lattice H_2O
310	Lattice H_2O
266	A_{1g} v_4 (UCl)
230	E_g v_{11} (UO_2^{2+})
219	E_g v_{11} (UO_2^{2+})

Figure S5. Fitted Raman spectrum of $K_2[UO_2Cl_4]$ after 50 kGy of γ radiation.[3]

Raman Spectra of $Rb_2[UO_2Cl_4]$ before and after γ radiation

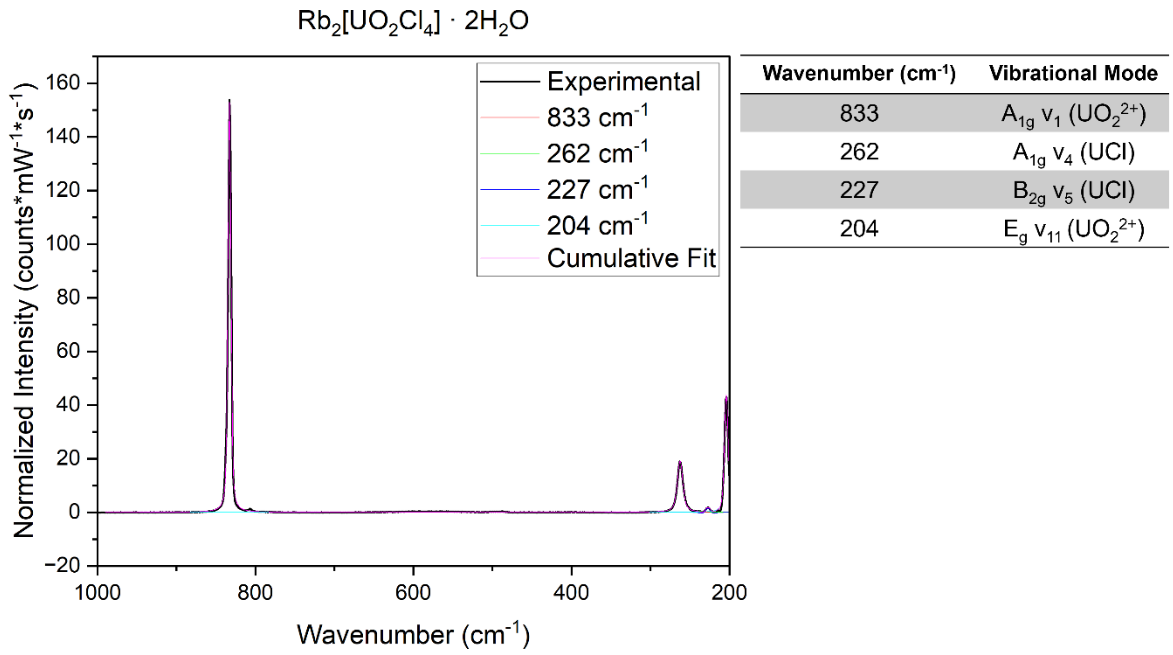

Wavenumber (cm^{-1})	Vibrational Mode
833	A_{1g} v_1 (UO_2^{2+})
262	A_{1g} v_4 (UCl)
227	B_{2g} v_5 (UCl)
204	E_g v_{11} (UO_2^{2+})

Figure S6. Fitted Raman spectrum of $Rb_2[UO_2Cl_4]$ solid pre-irradiation.[2]

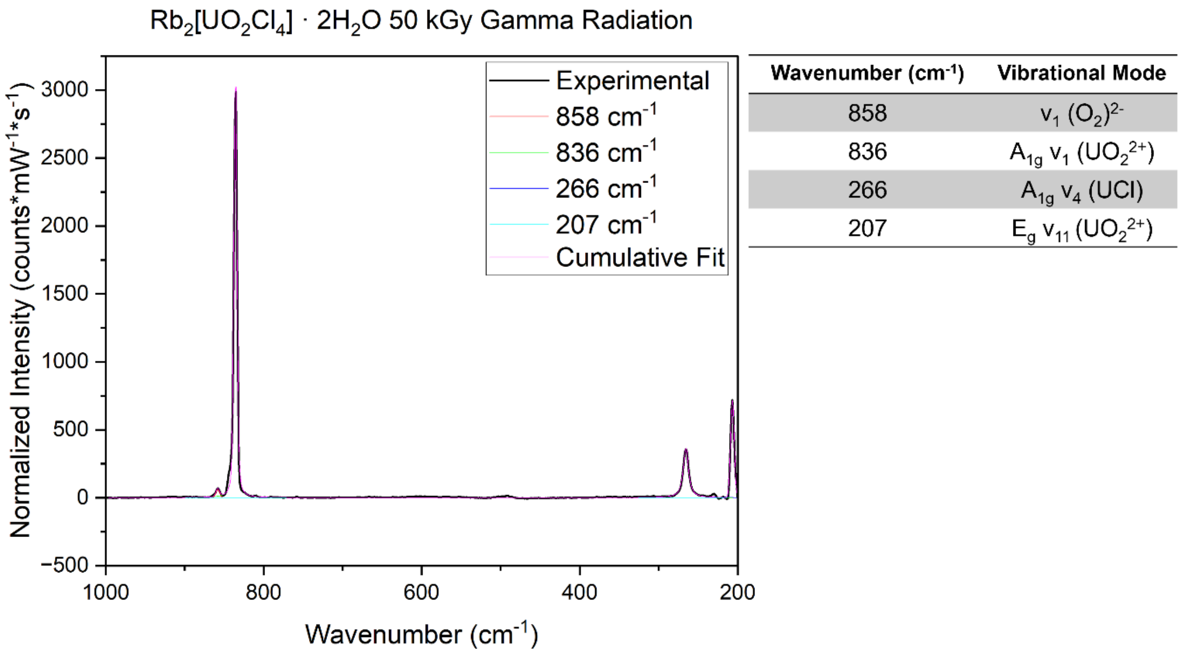

Wavenumber (cm^{-1})	Vibrational Mode
858	v_1 $(O_2)^{2-}$
836	A_{1g} v_1 (UO_2^{2+})
266	A_{1g} v_4 (UCl)
207	E_g v_{11} (UO_2^{2+})

Figure S7. Fitted Raman spectrum of $Rb_2[UO_2Cl_4]$ after 50 kGy of γ radiation.

Raman Spectra of $Rb_2[UO_2Cl_4]$ before and after γ radiation

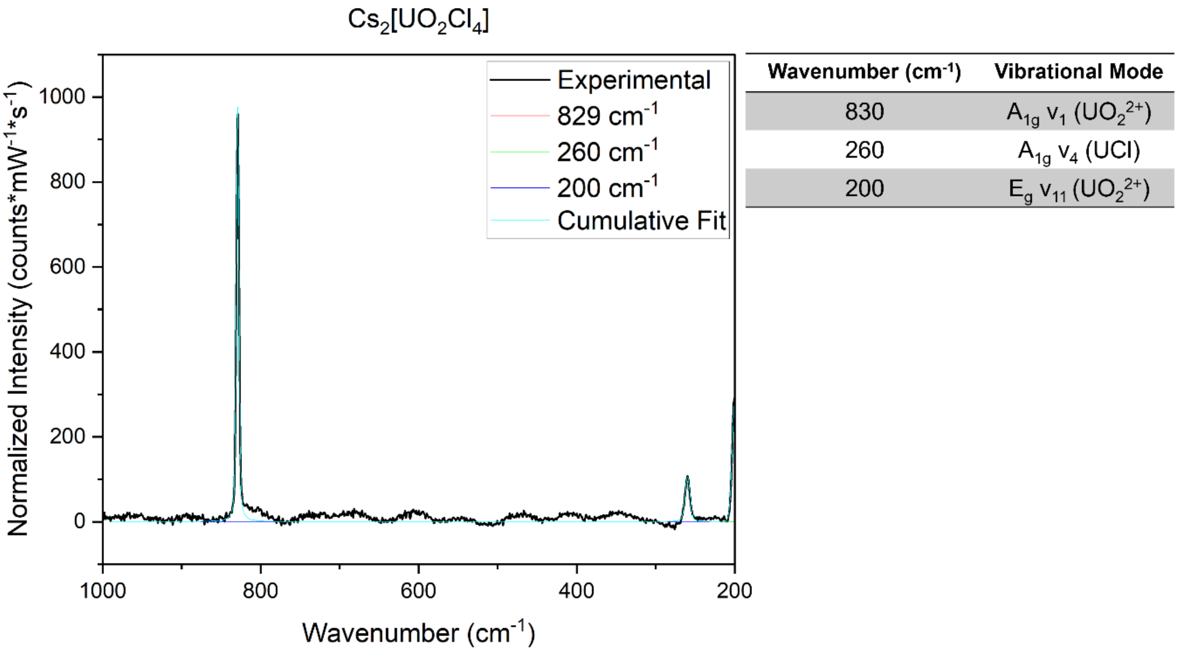

Wavenumber (cm^{-1})	Vibrational Mode
830	A_{1g} v_1 (UO_2^{2+})
260	A_{1g} v_4 (UCl)
200	E_g v_{11} (UO_2^{2+})

Figure S8. Fitted Raman spectrum of $Cs_2[UO_2Cl_4]$ solid pre-irradiation.[2]

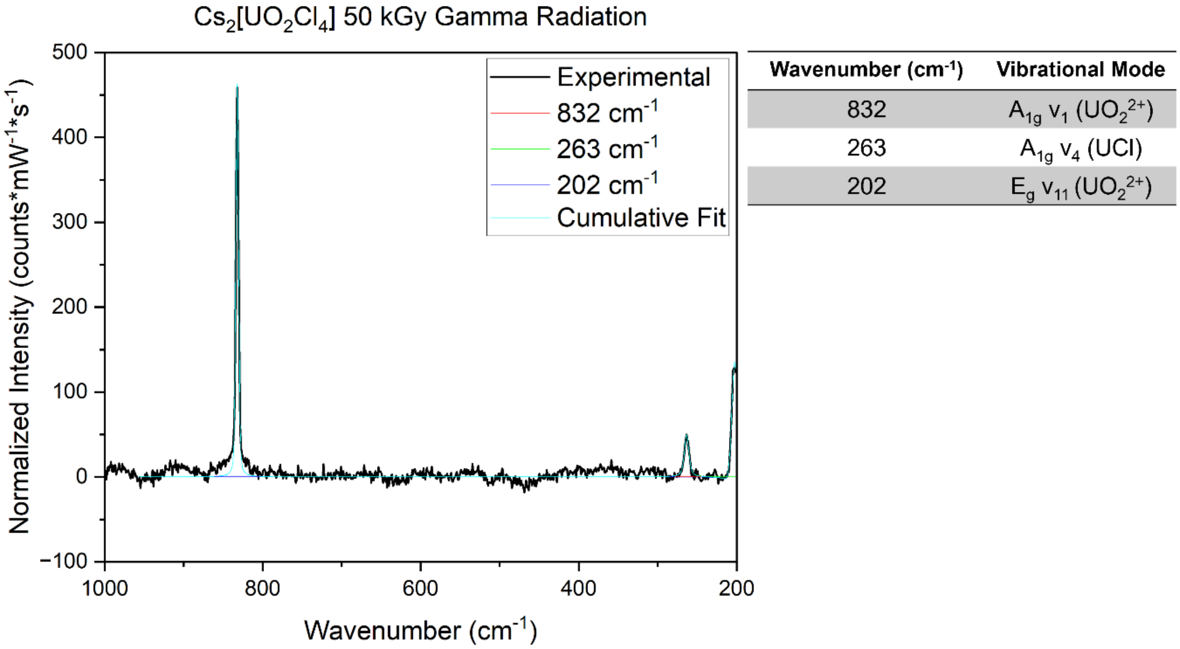

Wavenumber (cm^{-1})	Vibrational Mode
832	A_{1g} v_1 (UO_2^{2+})
263	A_{1g} v_4 (UCl)
202	E_g v_{11} (UO_2^{2+})

Figure S9. Fitted Raman spectrum of $Cs_2[UO_2Cl_4]$ after 50 kGy of γ radiation.

Mn(II) Impurity

EPR

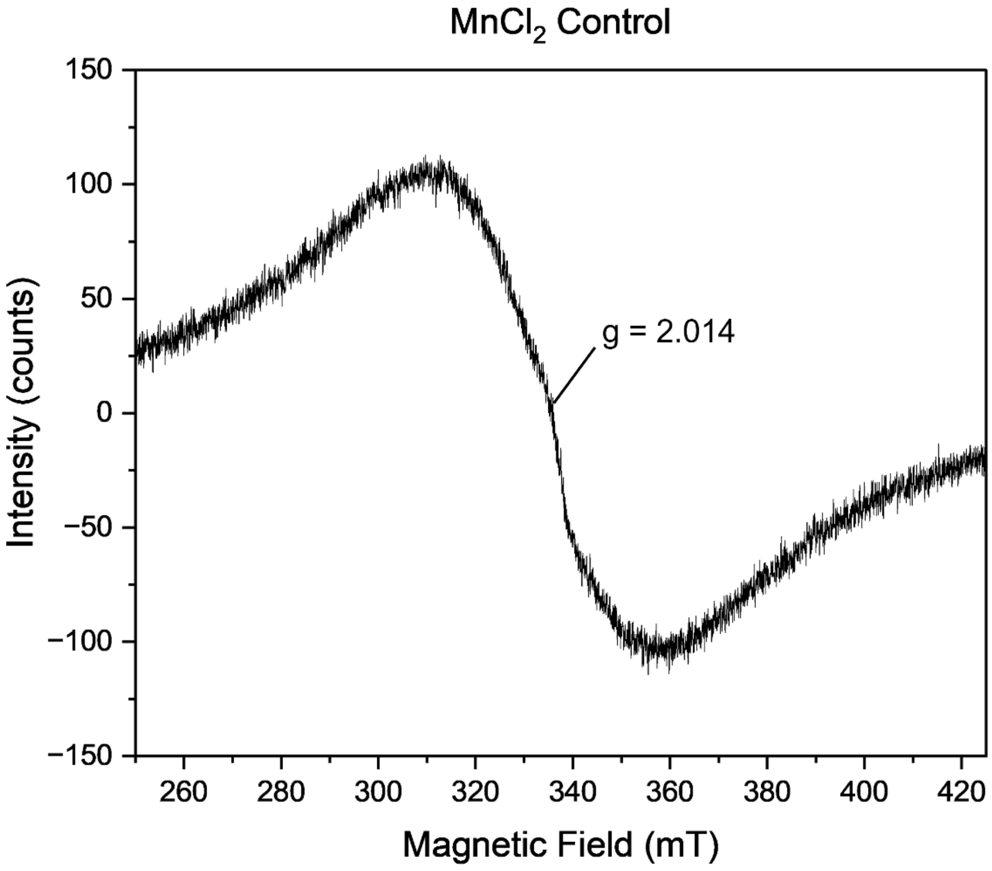

Figure S10. EPR spectrum of anhydrous $MnCl_2$ solid.

ICP-MS

Table S1. Summary of Mn-55 composition present in $Cs_2[UO_2Cl_4]$ dissolved in 3% nitric acid.

	Avg Intensity $Cs_2UO_2Cl_4$	Concentration (ppb)
Mn55-(MR)	1621700	100

Table S2. Summary of regression statistics for Mn-55 ICP-MS.

Regression Statistics	
Multiple R	0.996
R^2	0.993
Adjusted R^2	0.986
Standard Error	4259.81
Observations	3

Table S3. ANOVA of Mn-55 ICP-MS.

	df	*SS*	*MS*	*F*	*Significance F*
Regression	1	2613783818	2613783818	144.0414941	0.052921763
Residual	1	18146047.66	18146047.66		
Total	2	2631929866			

Electron Paramagnetic Resonance (EPR) Spectra

Dose Studies

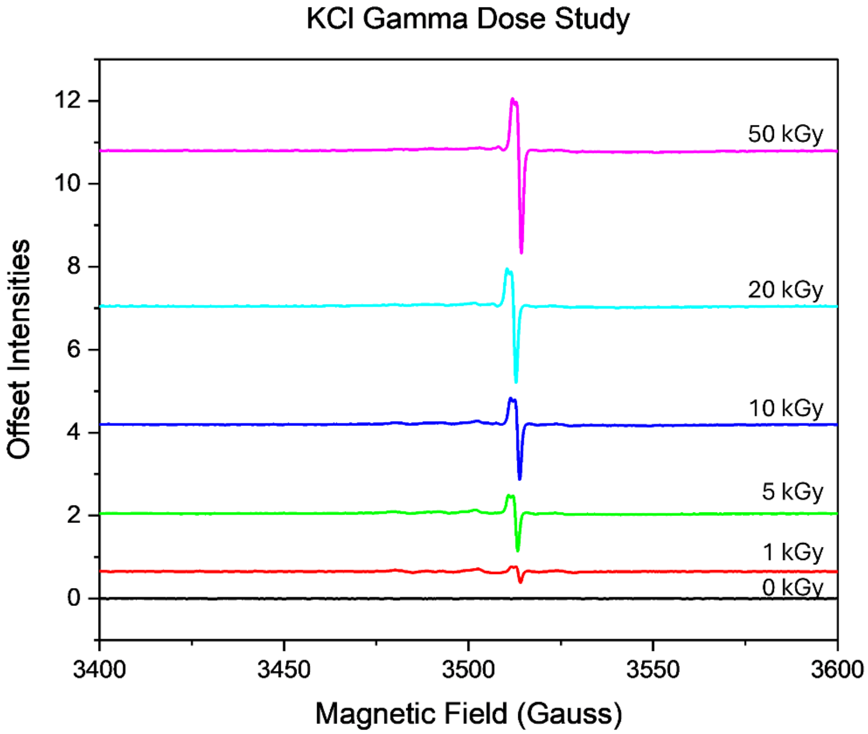

Figure S11. Stacked EPR spectra of KCl for γ-radiation dose study.

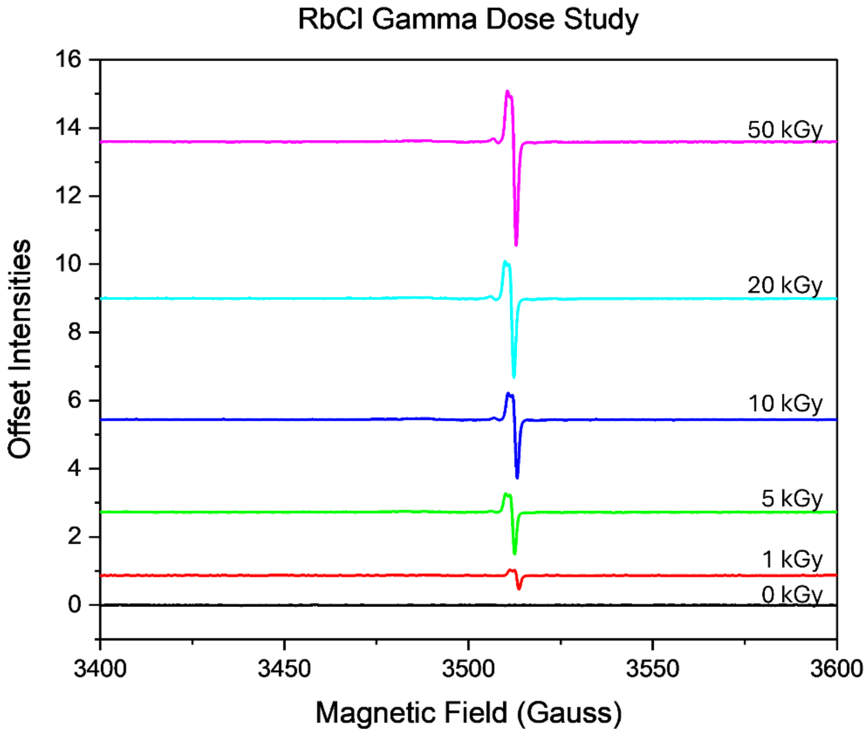

Figure S12. Stacked EPR spectra of RbCl for γ-radiation dose study.

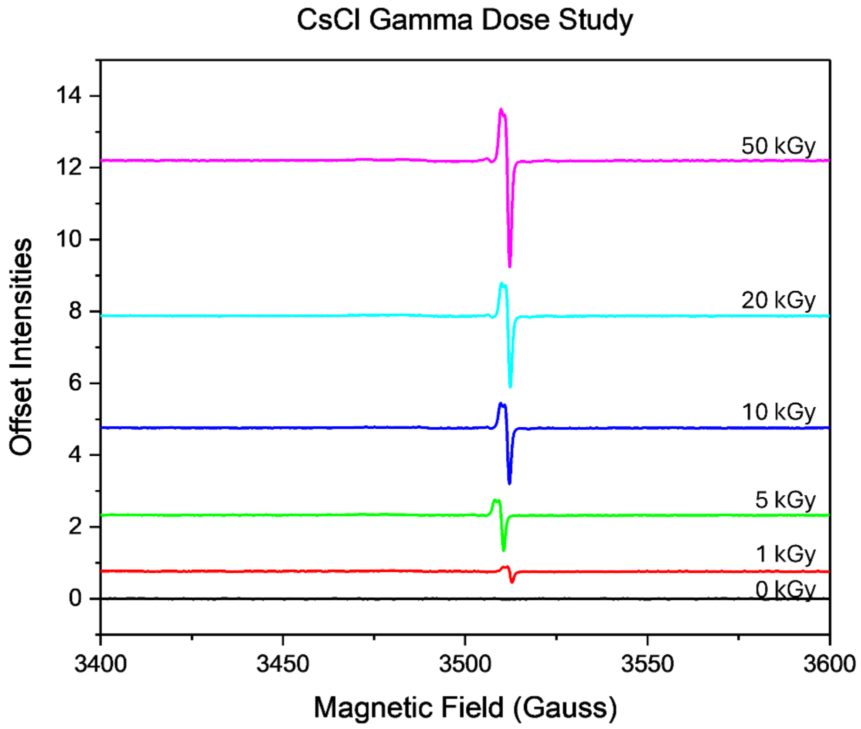

Figure S13. Stacked EPR spectra of CsCl for γ-radiation dose study.

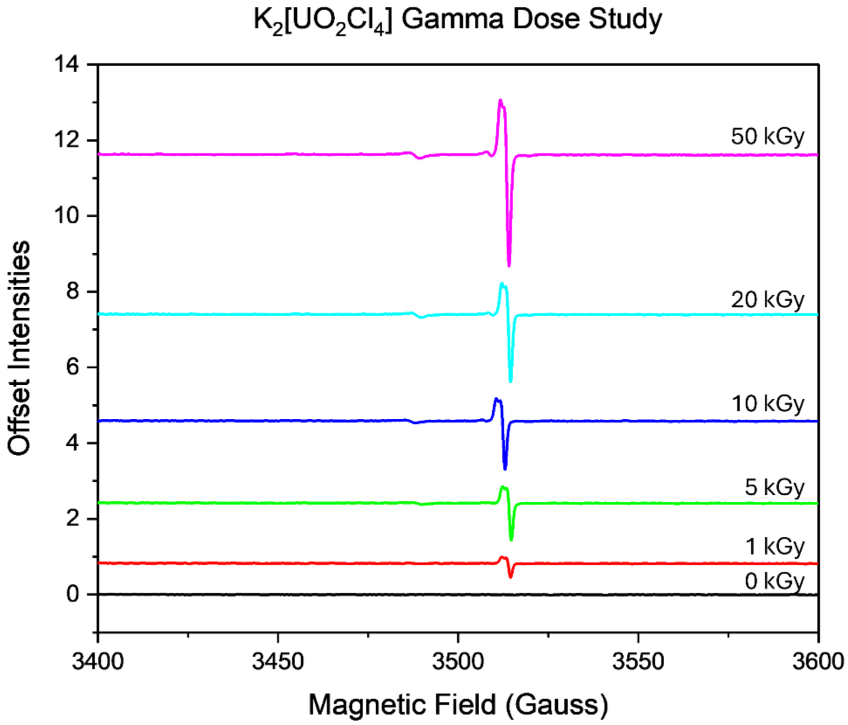

Figure S14. Stacked EPR spectra of $K_2[UO_2Cl_4]\cdot 2H_2O$ for γ-radiation dose study.

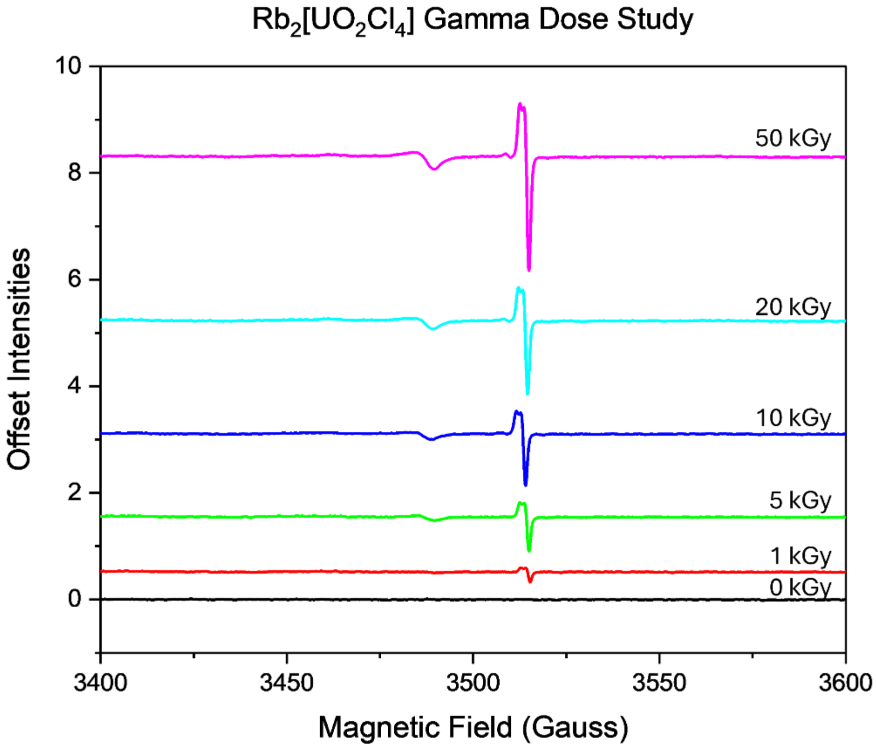

Figure S15. Stacked EPR spectra of $Rb_2[UO_2Cl_4]\cdot 2H_2O$ for γ-radiation dose study.

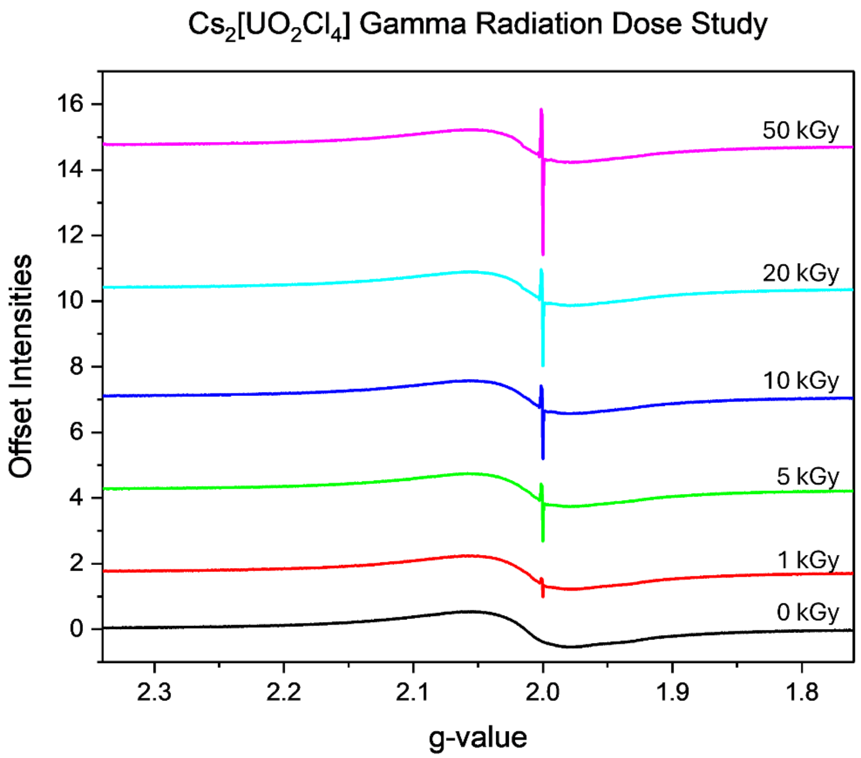

Figure S16. Stacked, full EPR spectra of $Cs_2[UO_2Cl_4]$ for γ-radiation dose study.

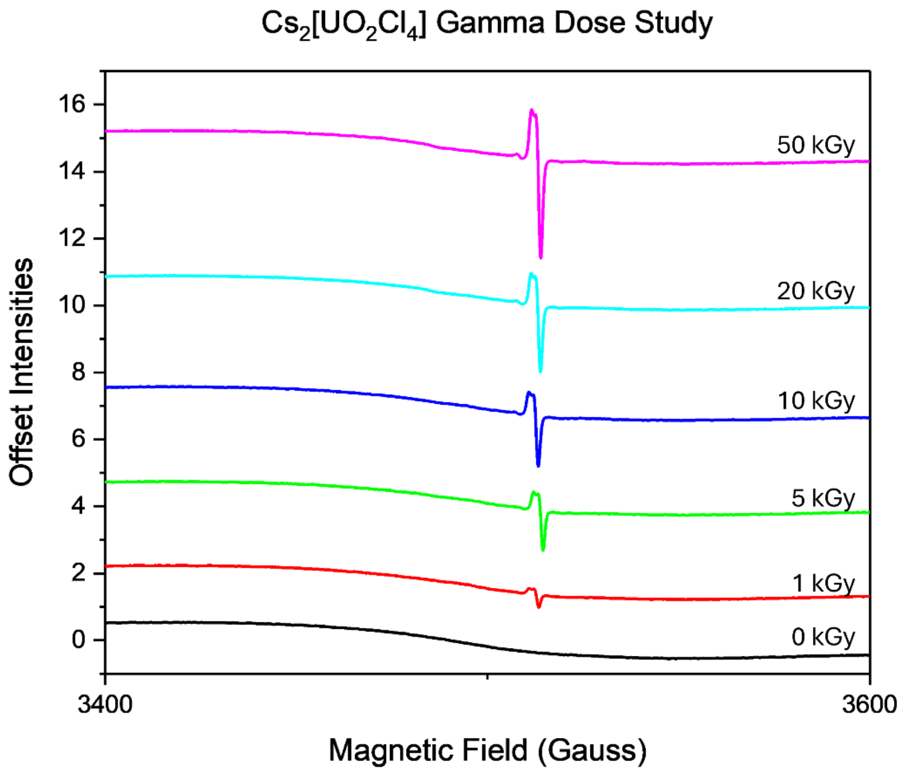

Figure S17. Stacked and enlarged EPR spectra of $Cs_2[UO_2Cl_4]$ for γ-radiation dose study.

Dose Studies 0-50 kGy

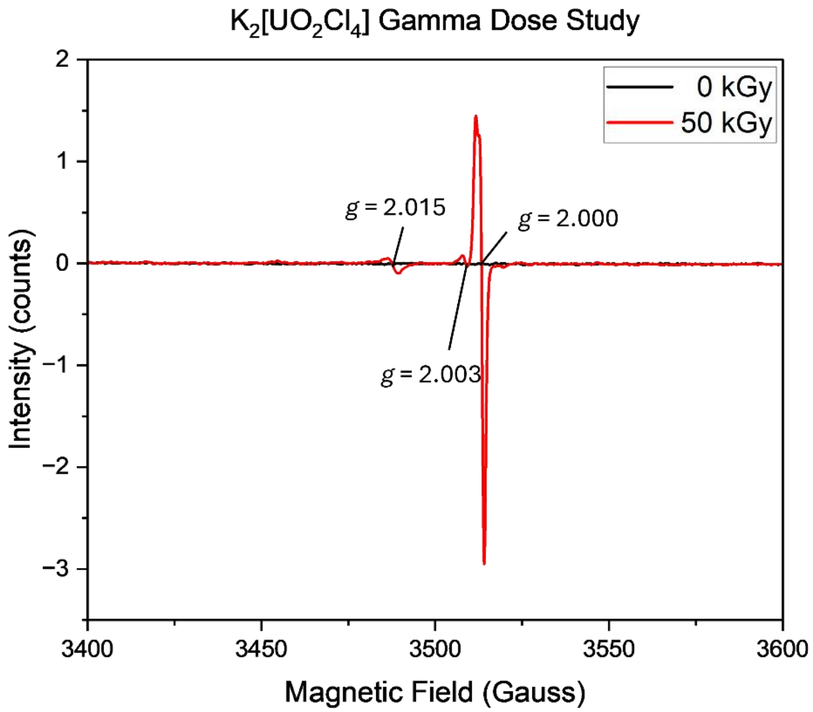

Figure S18. EPR spectra of $K_2[UO_2Cl_4]\cdot 2H_2O$ for γ-radiation dose study at 0 (black) and 50 kGy (red).

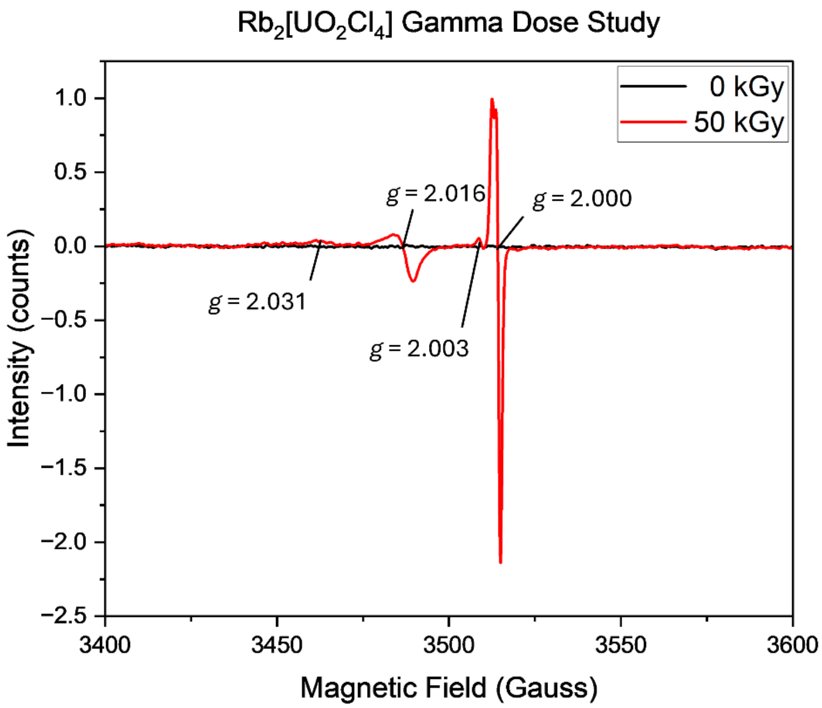

Figure S19. EPR spectra of $Rb_2[UO_2Cl_4]\cdot 2H_2O$ for γ-radiation dose study at 0 (black) and 50 kGy (red).

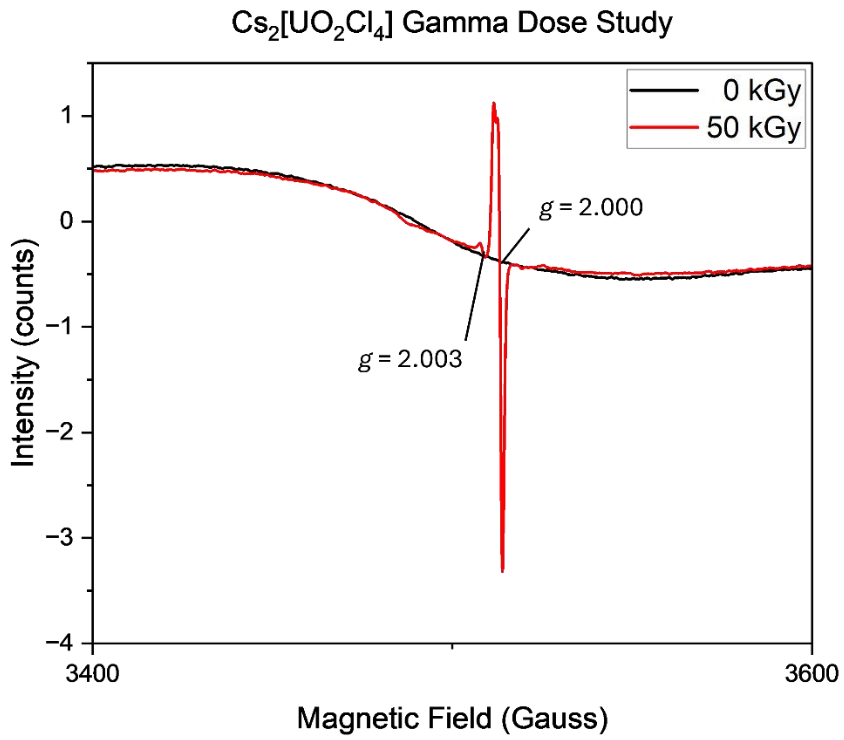

Figure S20. EPR spectra of $Cs_2[UO_2Cl_4]$ for γ-radiation dose study at 0 (black) and 50 kGy (red).

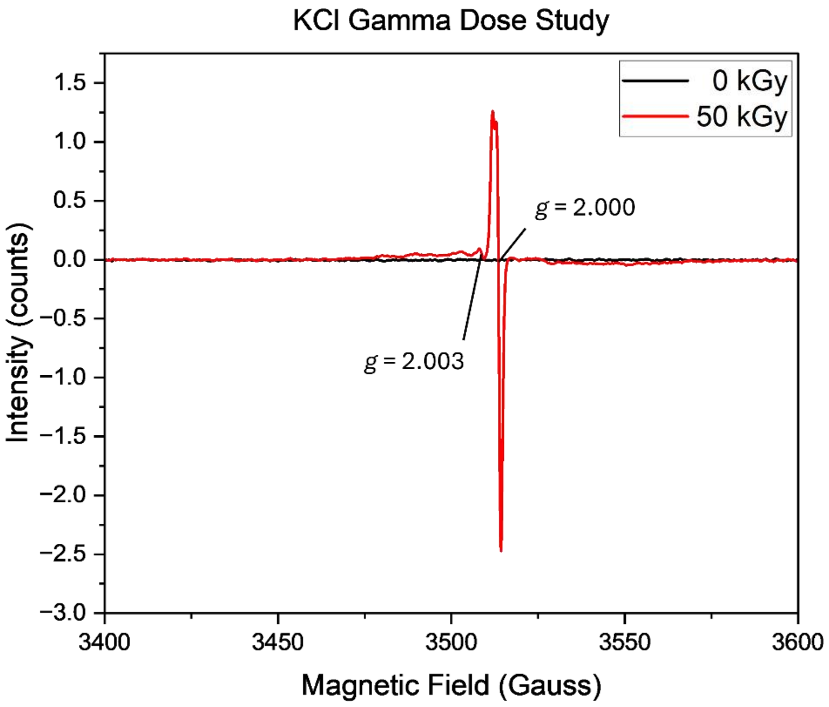

Figure S21. EPR spectra of KCl for γ-radiation dose study at 0 (black) and 50 kGy (red).

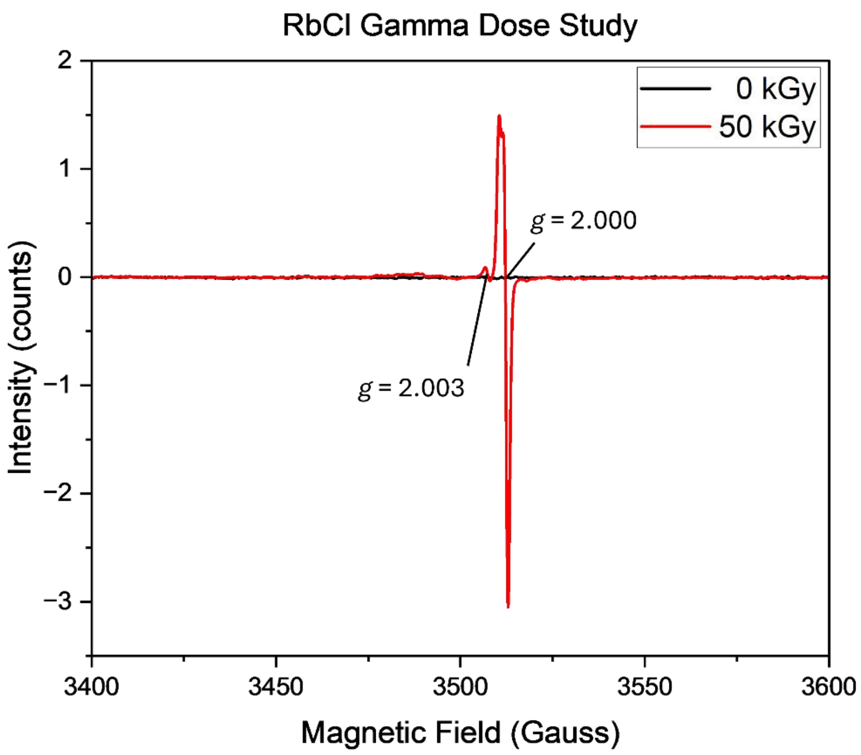

Figure S22. EPR spectra of RbCl for γ-radiation dose study at 0 (black) and 50 kGy (red).

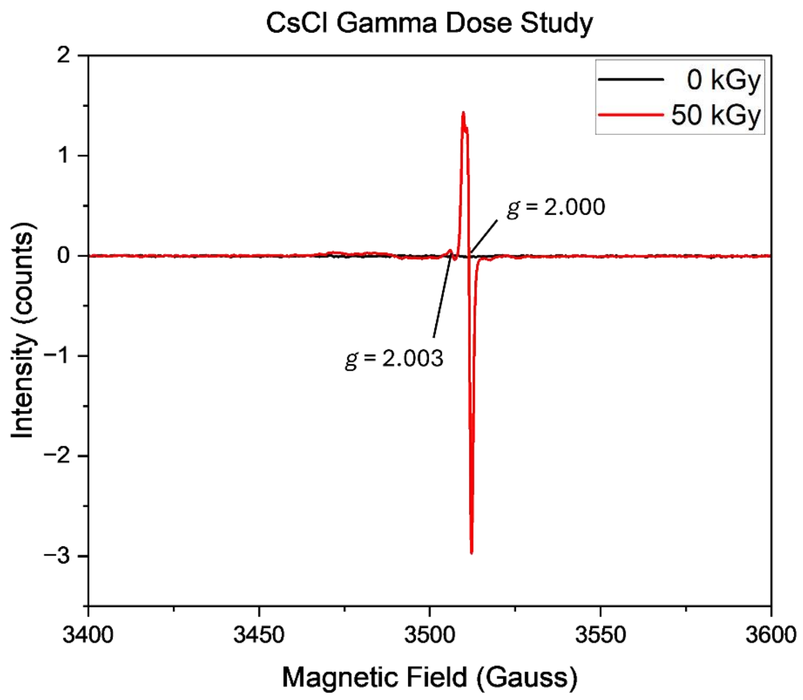

Figure S23. EPR spectra of CsCl for γ-radiation dose study at 0 (black) and 50 kGy (red).

DFT optimized bond lengths of the uranyl complexes

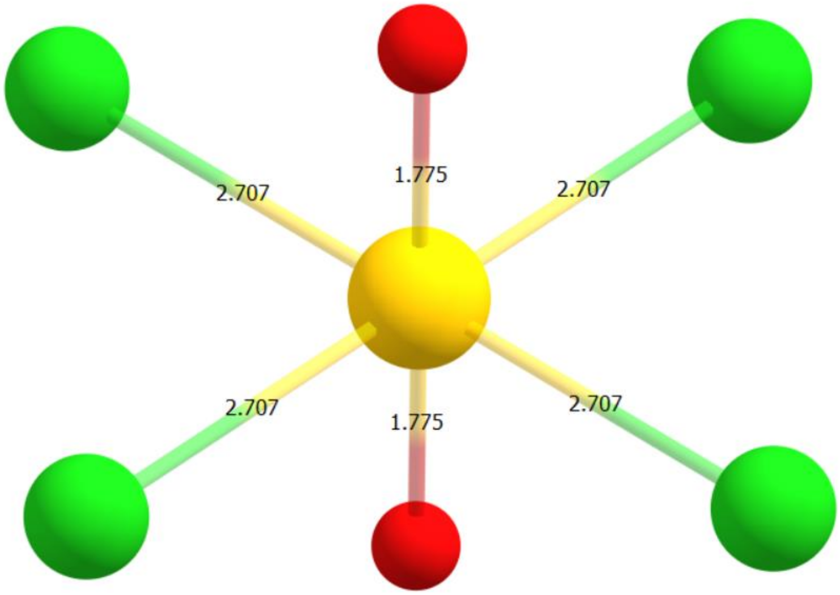

Figure S24. DFT optimized bond lengths of $[UO_2Cl_4]^{2-}$.

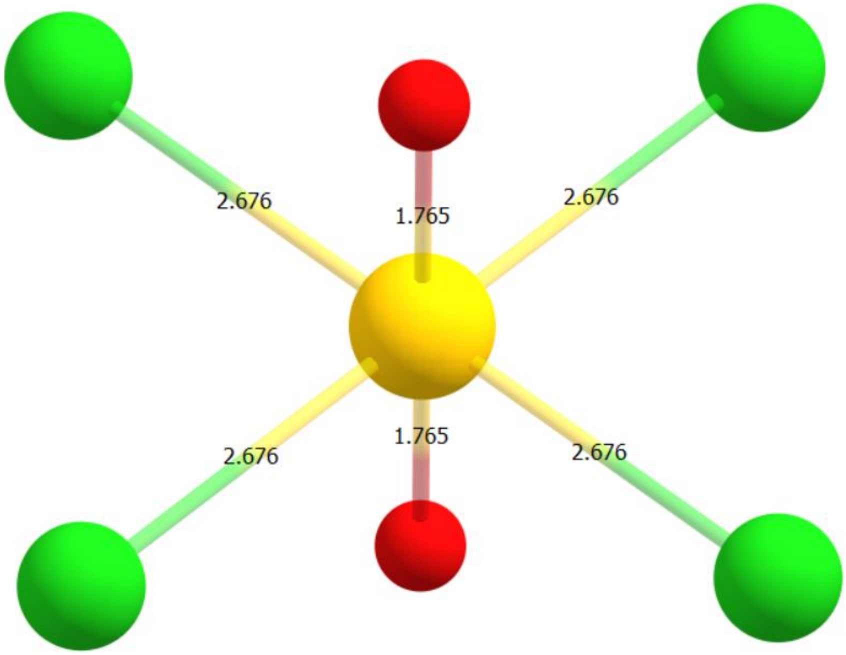

Figure S25. DFT optimized bond lengths of $[UO_2Cl_4]^{\cdot-}$

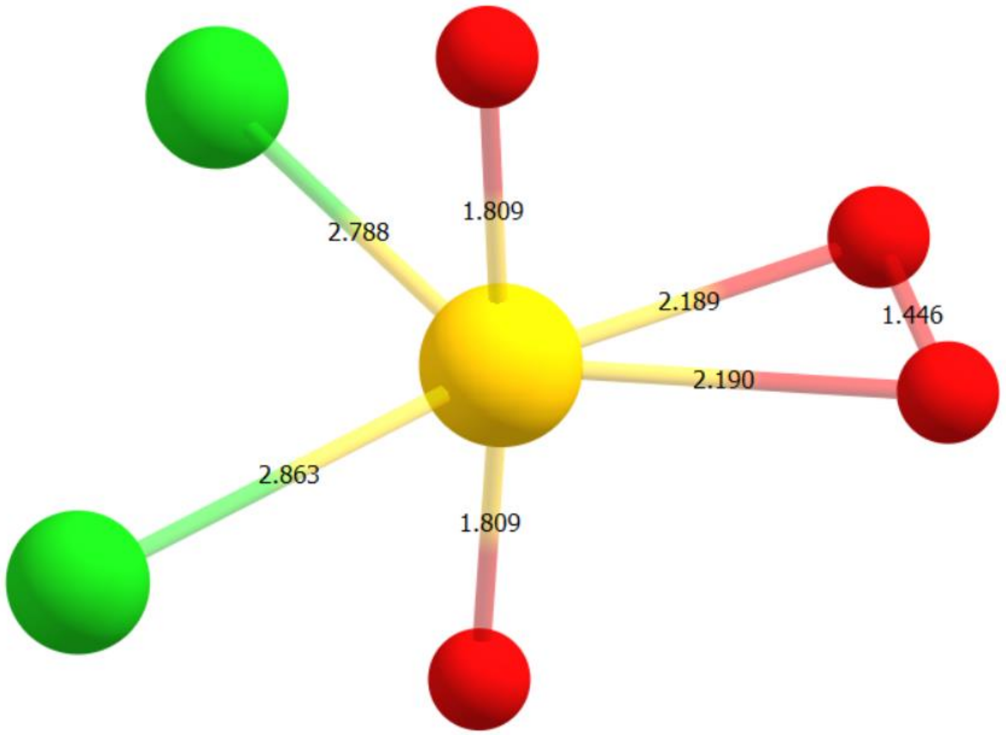

Figure S26. DFT optimized bond lengths of $[UO_2Cl_2(O_2)]^{2-}$

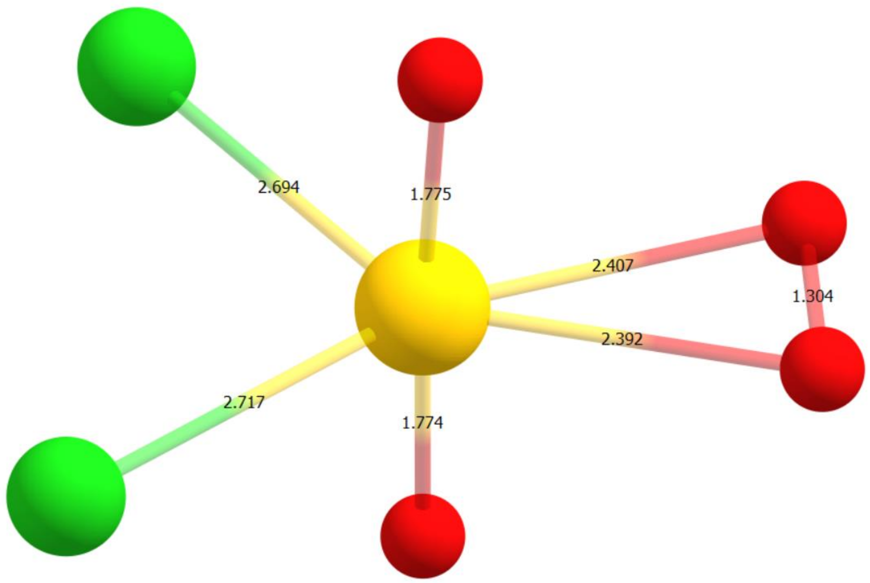

Figure S27. DFT optimized bond lengths of $[UO_2Cl_2(O_2)]^{\cdot-}$

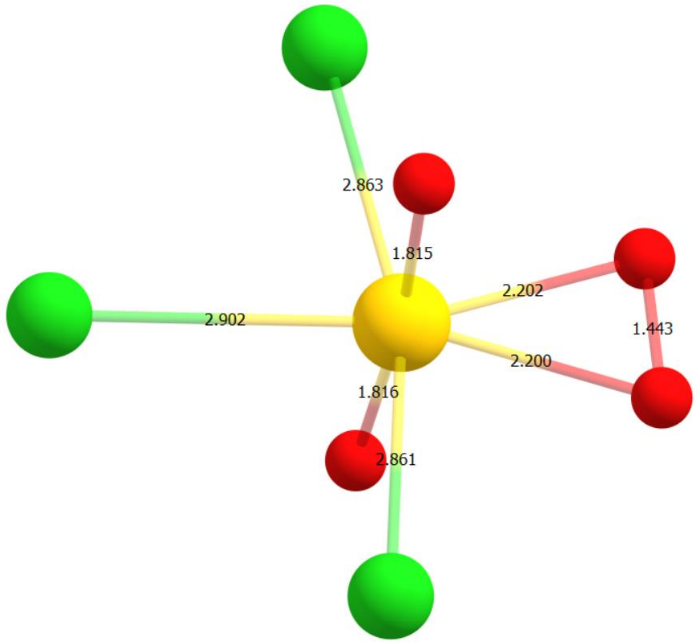

Figure S28. DFT optimized bond lengths of $[UO_2Cl_3(O_2)]^{3-}$

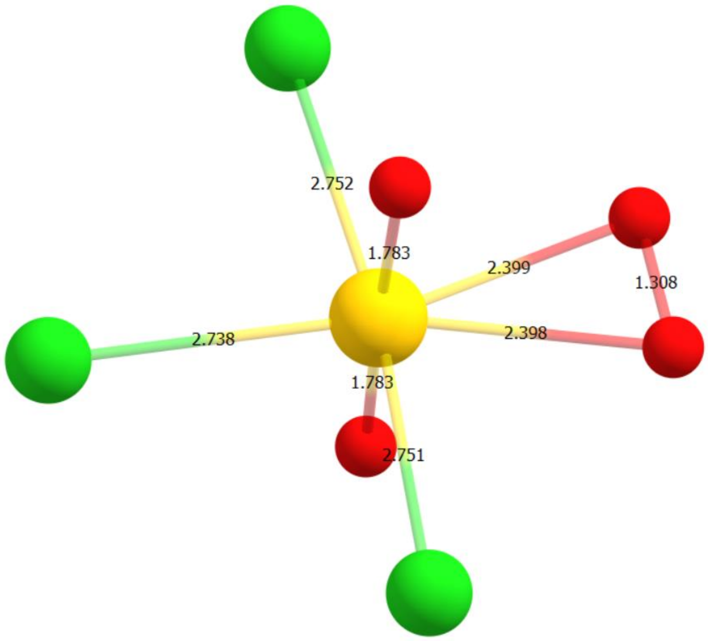

Figure S29. DFT optimized bond lengths of $[UO_2Cl_3(O_2)]^{\cdot 2-}$

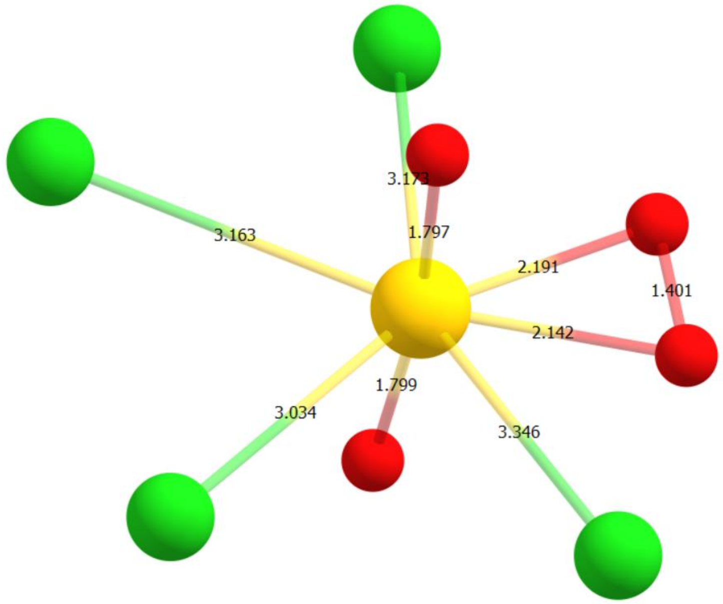

Figure S30. DFT optimized bond lengths of $[UO_2Cl_4(O_2)]^{4-}$

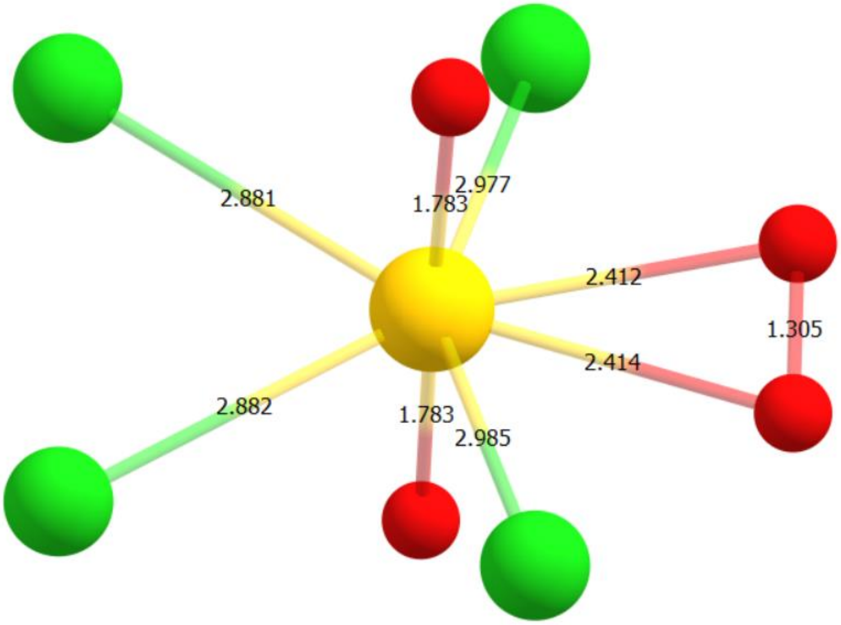

Figure S31. DFT optimized bond lengths of $[UO_2Cl_4(O_2)]^{\cdot 3-}$

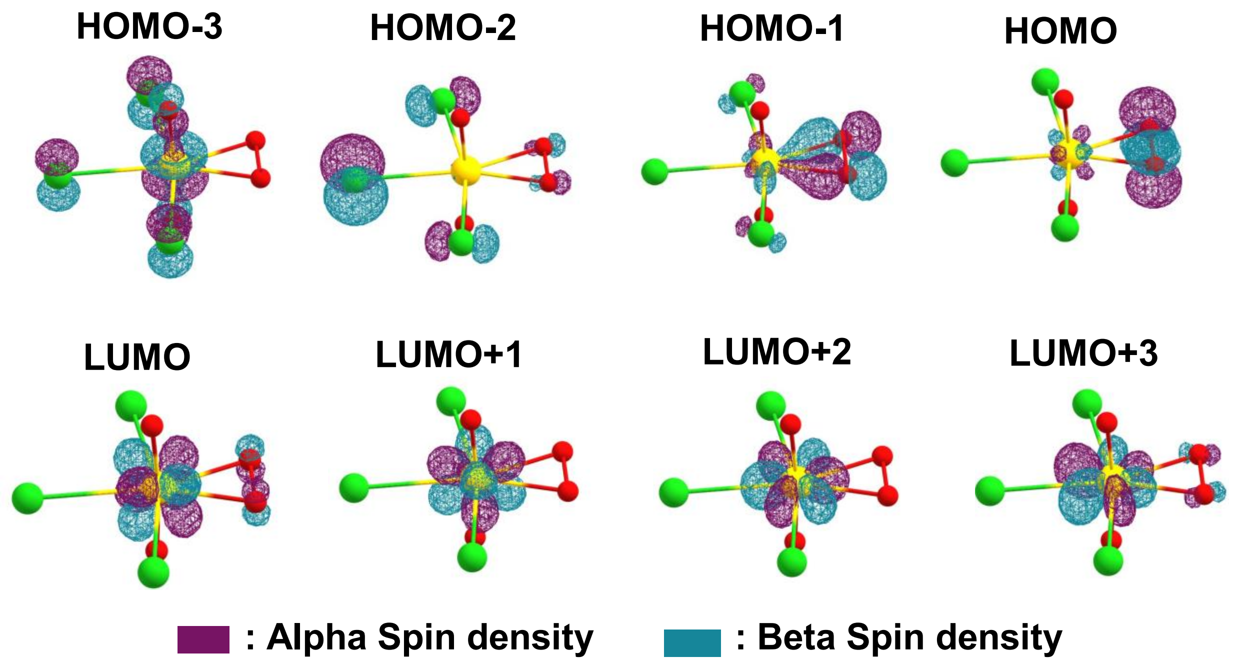

Figure S32. HOMO-3 to LUMO+3 molecular orbitals of $[UO_2Cl_3(O_2)]^{3-}$. The orbitals are visualized with counter values of 0.075.

Table S4. DFT calculated vibrational modes between spectral window of 700cm^{-1} to 1250 cm^{-1}

Compounds	Raman active modes	IR active modes
$[UO_2Cl_4]^{2-}$	UO_2^{2+} υ_1: 844 cm^{-1}	UO_2^{2+} υ_3: 903 cm^{-1}
$[UO_2Cl_4]^{\cdot-}$	UO_2^{2+} υ_1: 868 cm^{-1}	UO_2^{2+} υ_3: 941 cm^{-1}
$[UO_2Cl_2(O_2)]^{2-}$	UO_2^{2+} υ_1: 779 cm^{-1}<br>O_2^{2-} sym stretch: 907 cm^{-1}	UO_2^{2+} υ_3: 830 cm^{-1}
$[UO_2Cl_2(O_2)]^{\cdot-}$	UO_2^{2+} υ_1: 849 cm^{-1}<br>$O_2^{\cdot-}$ sym stretch: 1235 cm^{-1}	UO_2^{2+} υ_3: 910 cm^{-1}
$[UO_2Cl_3(O_2)]^{3-}$	UO_2^{2+} υ_1: 768 cm^{-1}<br>O_2^{2-} sym stretch: 913 cm^{-1}	UO_2^{2+} υ_3: 816 cm^{-1}
$[UO_2Cl_3(O_2)]^{2-\cdot}$	UO_2^{2+} υ_1: 829 cm^{-1}<br>$O_2^{\cdot-}$ sym stretch: 1231 cm^{-1}	UO_2^{2+} υ_3: 888 cm^{-1}
$[UO_2Cl_4(O_2)]^{4-}$	––	––
$[UO_2Cl_4(O_2)]^{3-\cdot}$	UO_2^{2+} υ_1: 822 cm^{-1}<br>$O_2^{\cdot-}$ sym stretch: 1225 cm^{-1}	UO_2^{2+} υ_3: 884 cm^{-1}

$ClO^{\bullet}_2$	-	-
H_2O_2	O_2^{2-} sym stretch: 950 cm^{-1}	-
HO_2^-	O_2^{2-} sym stretch: 828 cm^{-1}	-
O_2^{2-}	O_2^{2-} sym stretch: 756 cm^{-1}	-
$HO_2^{\cdot}$	$O_2^{\cdot-}$ sym stretch: 1181 cm^{-1}	-
$O_2^{\cdot-}$	$O_2^{\cdot-}$ sym stretch: 1191 cm^{-1}	-
$Cl_2^{\cdot-}$	-	-

DFT optimized geometries

Table S5. DFT optimized XYZ coordinates of $[UO_2Cl_4]^{2-}$

U	28.38000	21.62710	0.31690
O	28.38000	21.62710	2.08915
O	28.38000	21.62710	-1.45535
Cl	25.63266	21.62710	0.31690
Cl	28.38000	24.37444	0.31690
Cl	31.12734	21.62710	0.31690
Cl	28.38000	18.87976	0.31690

Table S6. DFT optimized XYZ coordinates of $[UO_2Cl_4]^{\cdot-}$

U	28.38000	21.62710	0.31690
O	28.38000	21.62710	2.08160
O	28.38000	21.62710	-1.44780
Cl	25.70370	21.62710	0.31690
Cl	28.38000	24.30340	0.31690
Cl	31.05630	21.62710	0.31690
Cl	28.38000	18.95080	0.31690

Table S7. DFT optimized XYZ coordinates of $[UO_2Cl_2(O_2)]^{2-}$

U	28.59905	21.77840	0.38280
O	28.49626	21.89791	2.18519
O	28.61657	21.82459	-1.42577
Cl	25.95413	20.89799	0.32383
Cl	28.39195	24.63328	0.31110
O	30.37616	20.50115	0.45525
O	29.19161	19.67144	0.43947

Table S8. DFT optimized XYZ coordinates of $[UO_2Cl_2(O_2)]^{\cdot -}$

U	28.49389	21.77001	0.36742
O	28.50099	21.78638	2.14178
O	28.57854	21.69740	-1.40423
Cl	25.83032	21.36750	0.31600
Cl	28.13683	24.46291	0.30752
O	30.52043	20.50418	0.47299
O	29.56473	19.61637	0.47040

Table S9. DFT optimized XYZ coordinates of $[UO_2Cl_3(O_2)]^{3-}$

U	28.49887	21.73792	0.39510
O	28.32288	21.74386	2.20182
O	28.48060	21.74241	-1.42032
Cl	25.60839	21.95342	0.26010
Cl	28.33820	24.59594	0.39848
Cl	27.93343	18.93389	0.37825
O	30.62354	22.30834	0.48302
O	30.51827	20.86874	0.48168

Table S10. DFT optimized XYZ coordinates of $[UO_2Cl_3(O_2)]^{\cdot 2-}$

U	28.33905	21.74899	0.38802
O	28.29094	21.75377	2.17035
O	28.46120	21.73985	-1.39071
Cl	25.61141	21.94182	0.25832
Cl	28.36421	24.50022	0.38118
Cl	27.98089	19.02003	0.37031
O	30.68304	22.24237	0.50049
O	30.59344	20.93747	0.50016

Table S11. DFT optimized XYZ coordinates of $[UO_2Cl_4(O_2)]^{4-}$

U	28.60848	21.41724	0.42867
O	28.33554	21.53975	2.21743
O	28.62428	21.52340	-1.37855
Cl	25.52557	22.11068	0.00905
Cl	27.95059	24.41499	0.57812
Cl	31.30240	23.09498	0.07832
Cl	27.09406	18.66786	0.52005
O	30.70037	20.47377	0.50346
O	29.76028	19.44395	0.33083

Table S12. DFT optimized XYZ coordinates of $[UO_2Cl_4(O_2)]^{\cdot 3-}$

U	28.54946	21.50775	0.36151
O	28.49839	21.49042	2.14415
O	28.63029	21.48694	-1.41995

Cl	25.73880	21.96596	-0.07549
Cl	27.96284	24.31002	0.69092
Cl	31.03258	23.14044	0.07623
Cl	27.02287	18.96576	0.62984
O	30.65961	20.36680	0.62744
O	29.80673	19.45251	0.25272

Table S13. DFT optimized XYZ coordinates of $ClO_2^{\cdot}$

O	0.86596	0.07259	0.00000
O	0.06633	0.95308	0.00000
Cl	0.87931	3.07603	0.00000

Table S14. DFT optimized XYZ coordinates of $ClO_2^{\cdot}$

O	0.86596	0.07259	0.00000
O	0.06633	0.95308	0.00000
Cl	0.87931	3.07603	0.00000

Table S15. DFT optimized XYZ coordinates of $HO_2^{\cdot}$

O	0.95025	0.24900	0.00000
O	0.02392	1.19836	0.00000
H	0.51523	2.04425	0.00000

Table S16. DFT optimized XYZ coordinates of $HO^{\cdot}$

O	0.49202	-0.04796	0.00000
H	0.16598	0.87296	0.00000

Table S17. DFT optimized XYZ coordinates of $H_2O^{+\cdot}$

O	8.21638	-0.01600	0.00000
H	9.22444	-0.02029	0.00000
H	7.87578	0.93278	0.00000

Table S18. DFT optimized XYZ coordinates of $O_2^{-\cdot}$

O	0.95314	0.22387	0.00000
O	0.03926	1.21453	0.00000

References

(1) Park, K.; Novotny, M.; Dalal, N.; Hill, S.; Rikvold, P. Effects of D-strain, g-strain, and dipolar interactions on EPR linewidths of the molecular magnets Fe 8 and Mn 12. *Physical Review B* **2001**, *65* (1), 014426.
(2) Schnaars, D. D.; Wilson, R. E. Structural and Vibrational Properties of U(VI)O2Cl42- and Pu(VI)O2Cl42- Complexes. *Inorganic Chemistry* **2013**, *52* (24), 14138-14147. DOI: 10.1021/ic401991n.
(3) Flint, C. D.; Tanner, P. A. Luminescence and absorption spectra of M 2UO2Cl4·xH2O. *Molecular Physics* **1981**, *44* (2), 411-425. DOI: 10.1080/00268978100102541.
